# A One‐Pot RPA‐CRISPR/Cas12a Assay for Rapid Genus‐Level Detection of *Babesia* spp. in Ticks and Livestock Blood Samples

**DOI:** 10.1155/tbed/9289663

**Published:** 2026-07-29

**Authors:** Wenxue Lu, Qiankun Yang, Peng Zhao, Yutong Cao, Zeqian Jing, Nan Zhang, Jianhua Li, Xin Li, Xiaocen Wang, Xu Zhang, Lili Cao, Pengtao Gong

**Affiliations:** ^1^ State Key Laboratory for Diagnosis and Treatment of Severe Zoonotic Infectious Diseases, Key Laboratory for Zoonosis Research of the Ministry of Education, and College of Veterinary Medicine, Jilin University, Changchun 130062, China, jlu.edu.cn; ^2^ College of Animal Science, Shanxi Agricultural University, Taiyuan, China, sxau.edu.cn; ^3^ Laboratory of Parasitology, Jilin Academy of Animal Husbandry and Veterinary Medicine, Changchun, China

**Keywords:** *Babesia* spp., CRISPR/Cas12a, one-pot detection, point-of-care testing, recombinase polymerase amplification

## Abstract

Babesiosis, a globally significant tick‐borne disease, poses substantial threats to livestock production and public health. Reported cases of human babesiosis in the United States increased from 1742 in 2014 to 3586 in 2023. In livestock, cattle babesiosis causes mortality, reduced meat and milk production, reproductive losses, and substantial control costs, with annual economic losses estimated at hundreds of millions of US dollars in several endemic countries. Rapid and sensitive detection methods are essential for early warning, surveillance, and control of this disease. In this study, we developed a closed‐tube, one‐pot assay for genus‐level detection of *Babesia* spp. associated with cattle and sheep, based on recombinase polymerase amplification (RPA) coupled with clustered regularly interspaced short palindromic repeats (CRISPR)/Cas12a. This format effectively minimizes cross‐contamination risks associated with repeated tube opening in conventional assays. A three‐channel signal readout system, including blue‐light fluorescence visualization, ultraviolet (UV) fluorescence visualization, and lateral flow strip (LFS) readout, was integrated to enable flexible endpoint detection under different laboratory and field conditions. The assay targets a conserved region of the *Babesia* 18S rRNA gene and enables genus‐level detection of *Babesia* spp. within 40 min at 37°C. The established RPA‐CRISPR/Cas12a platform exhibited high analytical sensitivity, with a limit of detection of 5 copies/μL for recombinant plasmid templates, high analytical specificity against the tested nontarget pathogens, and low equipment dependency. The detection limit of the LFS format reached 50 copies/μL. Field validation using 71 pooled tick samples and 53 clinical blood samples collected from cattle and sheep yielded positive rates of 15.49% and 9.43%, respectively, with 100% concordance between this assay and conventional polymerase chain reaction (PCR) for both specimen types. In conclusion, this one‐pot RPA‐CRISPR/Cas12a detection platform provides a rapid, sensitive, and field‐applicable molecular screening tool for genus‐level detection of *Babesia* spp. This assay may support early warning and preliminary field monitoring of babesiosis, particularly in resource‐limited settings. However, species‐level confirmation should be performed by sequencing or other species‐specific methods when epidemiological tracing or precise species identification is required.

## 1. Introduction

Babesiosis is a globally distributed tick‐borne zoonotic parasitic disease caused by intraerythrocytic protozoa of the genus *Babesia*, posing risks to both public health and the livestock industry [1]. Its transmission and prevalence are closely associated with the geographic distribution of competent tick vectors, and the disease has been reported across Europe, Asia, Africa, and other regions [2]. Clinical manifestations range from asymptomatic or mild infection to severe malaria‐like disease and even death [3, 4]. Severe babesiosis occurs more frequently in splenectomized individuals, the elderly, immunocompromised patients, and infants, and is commonly characterized by high fever, chills, hemolytic anemia, thrombocytopenia, and jaundice [5–7]. Surveillance data from the U.S. Centers for Disease Control and Prevention (CDC) showed that reported human babesiosis cases in the United States increased from 1742 in 2014 to 3586 in 2023 [8]. In addition, a nationwide hospitalization analysis in the United States identified 7818 babesiosis‐associated hospitalizations during 2010–2016, with an in‐hospital mortality rate of 1.6% [9]. In livestock, babesiosis affects cattle and sheep, causing high fever, anemia, hemoglobinuria, increased mortality, reduced production and reproductive performance, and increased prevention and control costs [10, 11]. Epidemiological surveys have reported widespread *Babesia* infection in livestock with seroprevalence in sheep ranging from 5.85% to 86.4% in Iran [12, 13]. In China, Yang et al. [14] investigated 2364 samples collected from 17 provinces (municipalities) and identified 589 positive cases, corresponding to an overall positivity rate of 24.92%. Therefore, the development of rapid, sensitive, and field‐deployable diagnostic methods is essential for the early warning, epidemiological surveillance, and control of babesiosis.

Currently, clinical diagnosis of *Babesia* infection primarily relies on microscopic examination, in which the morphology of parasites within red blood cells is directly observed on stained blood smears [15]. Microscopic examination of Giemsa‐stained thin blood smears is widely used for small‐scale clinical testing because of its simplicity [16]. However, its diagnostic sensitivity is influenced by parasite burden and microscopist expertise; thick blood smears can detect ~20–50 parasites/μL depending on the skill of the microscopist, whereas polymerase chain reaction (PCR) is more sensitive in cases with low‐level parasitemia [17]. Among serological techniques, the indirect fluorescent antibody test (IFAT) and enzyme‐linked immunosorbent assay (ELISA) are widely used, with diagnostic performance varying depending on antigen selection, cut‐off values, and infection stage. However, these techniques have several limitations: they may show cross‐reactivity with other apicomplexan parasites, potentially leading to false‐positive results, and exhibit lower detection efficacy during the early stages of infection, potentially resulting in missed diagnoses [18–20]. In recent years, molecular diagnostic technologies have been increasingly applied in *Babesia* detection. Among these, PCR is recognized as a sensitive and specific method for laboratory confirmation of this pathogen [21, 22]. However, its requirement for sophisticated instrumentation, along with complex operational procedures and a multistep workflow that generally requires thermal cycling followed by postamplification analysis, means that conventional PCR detection usually takes ~1.5–2 h or longer when DNA extraction and gel electrophoresis are included, which limits its use in point‐of‐care (POC) settings in veterinary clinical practice [23]. Given these equipment and workflow requirements, there is an urgent need to develop rapid and accurate on‐site diagnostic technologies to meet field‐based diagnostic needs.

Recombinase polymerase amplification (RPA) is an isothermal nucleic acid amplification technique that enables rapid and exponential amplification at a constant temperature of 37–42°C, with detectable products typically generated within 10–30 min through the coordinated action of recombinase enzymes and single‐stranded DNA (ssDNA)‐binding proteins [24]. Owing to its independence from thermocycling instrumentation, RPA offers high amplification efficiency, operational simplicity, and rapid signal generation, making it suitable for a broad range of molecular diagnostic applications. To date, RPA has been successfully applied to the detection of DNA and RNA targets from a wide spectrum of pathogenic organisms, including viruses, bacteria, and parasites [25]. These features reduce operational complexity and energy requirements, thereby providing a useful basis for the development of POC testing (POCT) platforms, particularly in resource‐limited settings [26–28]. However, the relatively high tolerance of RPA to primer‐template mismatches may increase the risk of nonspecific amplification [17]. In the RPA–clustered regularly interspaced short palindromic repeats (CRISPR)/Cas12a assay, this risk can be partially mitigated by the crRNA‐guided Cas12a recognition step, which provides an additional layer of sequence discrimination after amplification [29, 30]. Nonspecific RPA products would be expected to generate a positive signal only if they contain sequences sufficiently complementary to the crRNA target region to activate Cas12a. Therefore, rational primer/crRNA design and experimental specificity validation are essential for assay reliability. In this study, both RPA primer specificity and crRNA‐guided target recognition were considered during assay design and validation to minimize the potential impact of mismatch‐tolerant amplification on the overall assay specificity.

The CRISPR system represents a promising platform for nucleic acid detection [31], in which Cas12a functions as a key effector protein of class II, type V CRISPR systems [32]. The class II CRISPR system requires only a single effector protein, such as Cas12a, to mediate target recognition and cleavage, thereby simplifying the operation and facilitating broader application. Upon crRNA‐guided binding to and cleavage of the target DNA, Cas12a becomes activated and exhibits nonspecific trans‐cleavage activity toward the surrounding ssDNA [33]. Based on the trans‐cleavage activity of Cas12a, a closed‐tube, one‐pot RPA‐CRISPR/Cas12a format was adopted in this study, allowing target amplification and Cas12a‐mediated detection to occur in the same sealed reaction tube rather than as two manually performed sequential steps. This design avoids postamplification tube opening, thereby simplifying the workflow and reducing the risk of carryover contamination [30, 34]. However, in the one‐pot system, RPA amplification and Cas12a‐mediated cleavage may compete after the RPA and CRISPR/Cas12a components are combined within the closed tube. Once Cas12a‐crRNA recognizes the PAM‐containing target sequence within the RPA amplicon, Cas12a can cleave the amplicon in cis and activate trans‐cleavage of the ssDNA reporter. If cis‐cleavage occurs too rapidly before sufficient amplicons accumulate, it may reduce the availability of amplification templates and interfere with the RPA efficiency. Therefore, selection of an appropriate PAM–protospacer target site is important to ensure sufficient Cas12a activation for signal generation while avoiding excessive early cleavage of newly generated amplicons [35]. Appropriate PAM–protospacer selection and optimization of ribonucleoprotein (RNP) concentration, ssDNA reporter concentration, and RPA reaction volume are expected to help balance RPA amplification and Cas12a‐mediated detection in the one‐pot assay [36]. In addition, crRNA directs Cas12a to specifically recognize a target DNA sequence of ~18–25 nt, and mismatches within this region can reduce Cas12a activation and cleavage activity. This high sequence specificity enables crRNA‐mediated Cas12a recognition to serve as a secondary screening step for RPA amplicons, thereby reducing false‐positive signals [29]. By incorporating ssDNA reporter molecules labeled with a fluorophore–quencher pair, the presence of target nucleic acids triggers Cas12a activation, leading to reporter cleavage and fluorescence signal release—a mechanism widely employed in nucleic acid detection [35].

In this study, we developed a closed‐tube, one‐pot RPA‐CRISPR/Cas12a platform for the rapid detection of cattle‐ and sheep‐associated *Babesia* spp. This platform features simple operation, high analytical sensitivity under the tested conditions, and reduced dependence on complex instrumentation while showing potential for field deployment. In addition, by integrating RPA‐mediated target amplification with the trans‐cleavage activity of Cas12a, efficient signal amplification was achieved within a closed reaction system, thereby reducing postamplification handling and potential carryover contamination. Results can be visualized through three readout formats, including blue‐light fluorescence visualization, ultraviolet (UV) fluorescence visualization, and lateral flow strip (LFS) readout, with the entire workflow completed within 60 min. Overall, this platform provides a practical molecular tool that, after further simplification of sample processing, may support POC genus‐level screening and field surveillance of *Babesia* spp. associated with cattle and sheep.

## 2. Materials and Methods

### 2.1. Experimental Materials

The pMD‐18T plasmid was purchased from Takara Bio Inc. (Dalian, China). Competent *Escherichia coli* DH5α cells were purchased from Takara Bio Inc. (Dalian, China); the DNA Isothermal Rapid Amplification Kit (basic) was purchased from Amplification Future (Weifang, China). The HiScribe T7 Quick High Yield RNA Synthesis Kit was purchased from New England Biolabs (MA, USA). The Trans2K DNA Marker was purchased from TransGen Biotech (Beijing, China). The 2 × SanTaq PCR Master Mix used for PCR was purchased from Sangon Biotech (Shanghai, China). The fluorescence‐quenched ssDNA (FQ‐ssDNA) reporter molecule (5´‐6‐FAM‐TTATT‐BHQ1–3´) and the FITC/biotin dual‐labeled ssDNA (FB‐ssDNA) reporter molecule (5′‐6‐FAM‐TTATT‐Biotin‐3′) were synthesized by General Biol Co., Ltd. (Anhui, China). The fluorescence reader was purchased from Agilent Technologies Inc. (California, USA), and the 470 nm Blu‐ray instrument was purchased from TIANamp Co., Ltd. (Beijing, China). All oligonucleotides, including primers, were synthesized by Comate Bioscience (Jilin, China). CRISPR Single System Test Strips (FAM/FITC) were purchased from Changchun Runze Biological Co., Ltd. (Changchun, China). A green transilluminator (TIANGEN) was used as the blue light source. The UV ‐light source was a gel imager (Bio‐Rad, USA).

### 2.2. Sample Source and Nucleic Acid Extraction

*Babesia bigemina*, *Babesia motasi*, and *Babesia duncani* were provided by Hebei Agricultural University. These *Babesia* positive‐control materials were confirmed by PCR amplification and Sanger sequencing before use in the specificity assay. *Foot*‐*and-mouth disease virus* and *Brucella melitensis* were provided by the Lanzhou Veterinary Research Institute of the Chinese Academy of Agricultural Sciences. *Theileria annulata*, *Schistosoma japonicum*, *Trypanosoma evansi*, *Bluetongue virus*, *Leptospira*, *Toxoplasma gondii*, and *Neospora caninum* were maintained in our laboratory. Nucleic acids from these pathogens were extracted using the TIANamp Genomic DNA Kit according to the manufacturer’s instructions. For the clinical evaluation, a total of 71 tick sample pools were collected in May 2025. Pools 1–41 were obtained from Shanxi Province, China, while pools 42–71 were collected from Jilin Province, China. Each pool consisted of 4–6 individual ticks. According to the available sample information, the tick specimens used in this study were mainly adult ticks. The tick pools from Shanxi Province mainly contained *Dermacentor nuttalli* and *Ixodes persulcatus*, whereas those from Jilin Province mainly contained *Dermacentor nuttalli*, *Ixodes persulcatus*, and *Dermacentor silvarum*. In addition, 53 venous whole blood samples from cattle and sheep were collected from Shanxi Province, China, using EDTA anticoagulant tubes. Genomic DNA was extracted from all 71 tick sample pools and 53 blood samples using the TIANamp Genomic DNA Kit according to the manufacturer’s instructions. The DNA concentration was quantified with a NanoDrop 2000 spectrophotometer, and the samples were subsequently stored at −80°C for further analysis. All cattle and sheep blood samples, *Babesia*‐negative mouse whole blood used for the plasmid‐spiked blood matrix experiment, and tick samples used in this study were collected and/or used with ethical approval from the Animal Ethical Committee of Jilin University (Permit Number SY202407006).

### 2.3. Expression and Purification of Cas12a

In the gene cloning process, the huLbCpf1 coding gene was amplified using the plasmid 6His‐MBP‐TEV‐huLbCpf1 as a template. The gene was then digested with EcoRI and NotI restriction enzymes and cloned into the pET‐28a expression vector (Figure S1A). The constructed vector was subsequently transformed into *E. coli* DH5α competent cells. Positive clones were selected and validated by sequencing to confirm the accuracy of the recombinant plasmid sequence. The validated plasmid was then transformed into Rosetta (DE3)‐competent cells and verified by PCR. After verification, recombinant protein expression was induced. The glycerol stock containing the positive vector was inoculated into 500 mL of LB medium supplemented with 50 μg/mL kanamycin, and cultured at 37°C with shaking until the OD_600_ reached ~0.6. Subsequently, 0.3 mM IPTG was added to induce protein expression at 16°C and 140–150 rpm for 20–22 h to promote soluble protein expression. After expression, the bacterial culture was centrifuged at 12,000 × *g* and 4°C to collect the cell pellets. Cells were then lysed by ice‐cold sonication, and the supernatant containing the target protein was collected following centrifugation. The obtained supernatant was loaded onto a pre‐equilibrated histidine (HIS)‐agarose chromatography column for protein purification. The expression and purification of the recombinant protein were evaluated by SDS‐PAGE (Figure S1B), and its trans‐cleavage activity was confirmed using the activity assay (Figure S1C). Finally, the validated protein samples were stored at −80°C or in liquid nitrogen to maintain their long‐term stability.

### 2.4. Design and Screening of Primers for RPA Reaction

The 18S rRNA gene of *Babesia* species mainly infecting cattle and sheep was selected as the target gene. Full‐length sequences of clinical isolates from different *Babesia* spp. mainly infecting cattle and sheep were selected from the GenBank database, and alignment was performed using SnapGene software to obtain the highly conserved 18S rRNA sequence (176 bp). Subsequently, based on this sequence, five pairs of RPA primers were designed using Primer 6.0, following the primer design principles for RPA (Table S1). All RPA reactions were performed using the Amplification Future DNA Isothermal Rapid Amplification Kit (Basic Type), with a total reaction volume of 50 μL. The standard reaction mixture included 0.50 μM each of the forward and reverse primers, 29.5 μL rehydration buffer, 5 μL of extracted genomic template DNA, and nuclease‐free water to adjust the volume. Prior to reaction initiation, all components except magnesium acetate were thoroughly mixed and briefly centrifuged to ensure complete dissolution of the lyophilized reaction pellet. Finally, 2.5 μL of 280 mM magnesium acetate was added to the tube cap, followed by centrifugation to initiate the amplification reaction. The reaction was performed at a constant temperature of 37°C for 30 min. After amplification, the products were purified using the phenol‐chloroform method and analyzed by 2% agarose gel electrophoresis to evaluate the results and select the primer pair with the best amplification performance.

### 2.5. Design and In Vitro Transcription of crRNA for the CRISPR‐Cas12a System

Based on the target gene sequence and the canonical TTTV PAM preference of LbCas12a, crRNA target sites were designed. To cover additional potential target loci within the conserved regions, a subset of crRNAs targeting suboptimal PAM sequences was also designed (Table S2). Each crRNA template comprised a T7 promoter, an LbCas12a‐specific crRNA scaffold, and the corresponding target sequence. Complementary oligonucleotides were annealed to generate double‐stranded DNA (dsDNA) templates. Annealing was performed in 10× NE Buffer r3.1 by heating at 95°C for 5 min, followed by gradual cooling to 25°C (1°C per 90 s). crRNAs were synthesized by in vitro transcription using the HiScribe T7 Quick High Yield RNA Synthesis Kit at 37°C for 4 h. Residual DNA templates were removed by DNase I digestion, and crRNAs were purified using the miRNeasy Mini Kit. Purified crRNAs were quantified by NanoDrop spectrophotometry (Figure S2), diluted to 8000 nM with nuclease‐free water, and stored at −80°C or in liquid nitrogen until use.

### 2.6. Construction of Standard Positive Recombinant Plasmid *Babesia*‐18S rRNA

Multiple homologous gene sequences of *Babesia* spp. that infect cattle and sheep, including *Babesia bigemina* (MH194385.1), *Babesia bovis* (HQ264112.1), *Babesia divergens* (U16370.1), *Babesia ovata* (XR_003751973.1), *Babesia motasi* (AY260179.1), *Babesia ovis* (AY260178.1), and *Babesia crassa* (AY260176.1) [10, 37] were retrieved from GenBank. These sequences were subjected to sequence alignment using the SnapGene software (Figure S3). A highly conserved region within the 18S rRNA gene was identified as the target fragment for primer design to enable broad‐range, genus‐level detection of *Babesia* spp. rather than species‐level discrimination. PCR amplification was performed using Taq DNA polymerase with the *Babesia*‐F/R primer pair designed for this region (Table S2). The amplified product was verified for specificity by 2% agarose gel electrophoresis, followed by purification using a DNA purification kit to recover the target fragment. The purity (OD_260_/OD_280_ = 1.8–2.0) and concentration of the purified product were quantified using a NanoDrop spectrophotometer. The target gene fragment was then ligated into the pMD‐18T vector at a molar ratio of 1:4 (vector:fragment) with the addition of the Solution I ligation reagent. The ligation reaction (5 μL) was incubated overnight at 4°C. The ligation product was subsequently transformed into *E. coli* DH5α competent cells, and the recombinant plasmid was verified through positive clone screening and sequencing. The correctly constructed plasmid was stored at −20°C for future use.

### 2.7. Establish a One‐Tube RPA‐CRISPR/Cas12a Detection System Based on 18S rRNA

The detection platform employed a closed‐tube, one‐pot design for the genus‐level detection of *Babesia* spp. The entire reaction system consisted of two modules, the RPA mixture and the CRISPR/Cas12a detection mixture, which were spatially separated during sample loading and combined by brief centrifugation to initiate the reaction. The RPA mixture was prepared following the protocol described in Section 2.4, with template DNA and magnesium acetate being omitted during preparation. A 20 μL aliquot of the RPA mixture was dispensed into the bottom of the reaction tube. For the CRISPR/Cas12a detection module, 25 μL of the detection mixture was pre‐loaded onto the inner surface of the tube cap. This mixture contained 300 nM RNP complex (Cas12a‐crRNA complex), along with either 500 nM FQ‐ssDNA reporter for fluorescence detection or 200 nM FB‐ssDNA reporter for lateral flow detection, with deionized water added to the final volume. Subsequently, 2.5 μL of template DNA and 2.5 μL of magnesium acetate were added to the inner wall of the reaction tube without contacting the RPA mixture at the tube bottom before centrifugation. Brief centrifugation was performed to bring all components (the cap‐side CRISPR/Cas12a mixture, the wall‐side template DNA and magnesium acetate, and the bottom RPA mixture) to the bottom of the tube for thorough mixing. Following centrifugation, RPA amplification was initiated upon activation by magnesium acetate, whereas Cas12a‐mediated reporter cleavage occurred after target recognition within the same reaction system. Therefore, RPA amplification and Cas12a‐mediated signal generation were not manually performed as separate sequential steps but instead occurred as temporally overlapping processes during incubation. As RPA amplicons accumulated, Cas12a activation and reporter cleavage generated increasing fluorescence or LFS signals. The reaction was then incubated at 37°C for 40 min, during which fluorescence signals were collected every 30 s using an AriaDx Real‐Time PCR System (Agilent Technologies, Santa Clara, CA, USA). After the reaction, the final product was visualized using a 470 nm blue‐light transilluminator or a 365 nm UV‐light gel imaging system, or alternatively, the reaction mixture was applied to a CRISPR/Cas12a‐specific test strip, and the results were visually observed. Specifically, the endpoint fluorescence positivity threshold was defined as the mean fluorescence intensity of the negative controls plus three standard deviations (mean + 3 SD). Samples with endpoint fluorescence signals above this threshold were considered positive, whereas samples with signals at or below this threshold were considered negative. This threshold‐setting strategy was uniformly applied to all sample types, including plasmid standards, plasmid‐spiked blood samples, pooled tick samples, and clinical blood samples. For visual fluorescence readouts, samples exhibiting clear green fluorescence under a blue‐light transilluminator or clear white fluorescence under a UV transilluminator were judged as positive; colorless and transparent samples were judged as negative. For LFS detection, the presence of both the control line and the test line was interpreted as positive, while the presence of only the control line was interpreted as negative.

### 2.8. Preparation of Plasmid‐Spiked Blood Matrix

*Babesia*‐negative mouse whole blood was used as the blood matrix for the spiking experiment. Fresh blood was collected and used immediately without any anticoagulant treatment. The *Babesia*‐negative status of the blood sample was confirmed by conventional PCR. The *Babesia*‐18S rRNA recombinant plasmid was 10‐fold serially diluted with nuclease‐free water from 5 × 10^4^ to 5 × 10^0^ copies/μL. For each reaction, 1 μL of the diluted recombinant plasmid was mixed with 1 μL of mouse whole blood to generate the plasmid‐spiked blood matrix. No DNA extraction was performed for these simulated blood samples; instead, the plasmid‐spiked whole–blood mixture was directly used as the template for the RPA‐CRISPR/Cas12a assay, which was performed at 37°C for 40 min.

### 2.9. PCR‐Based Detection of *Babesia* spp. in Pooled Tick and Blood Samples

To evaluate the performance of the developed detection method, a total of 71 pooled clinical tick samples and 53 clinical blood samples were analyzed. All samples were tested in parallel by both the established RPA‐CRISPR/Cas12a assay and PCR analysis. The primer pair designated as PCR‐B‐F/R was used for PCR amplification (Table S2) [38]. Each 25 μL PCR reaction mixture consisted of 12.5 μL of 2× SanTaq PCR Master Mix (Sangon Biotech Co., Ltd.), 1 μL each of the forward and reverse primers (10 μmol/L), 2 μL of template DNA, and nuclease‐free water added to a final volume of 25 μL. The reaction conditions were as follows: initial denaturation for 5 min at 94°C; 35 cycles of 30 s at 94°C, 30 s at 55°C, and 22 s at 72°C; and a final extension of 10 min at 72°C. The amplification products were separated on a 1.5% agarose gel, stained with ethidium bromide, and visualized under UV illumination; the presence of a 452‐bp band was considered indicative of a positive result in both tick and blood samples. Cohen’s kappa value was calculated as *κ* = (*P*_o_ − *P*_*e*_)/(1–*P*_*e*_), where *P*_o_ was the observed agreement and *P*_*e*_ was the expected agreement by chance.

### 2.10. Statistical Analysis

Data were analyzed using GraphPad Prism software (Version 10). Data were expressed as the mean ± SD. Comparisons between two groups were performed using an unpaired two‐tailed *t*‐test, while comparisons among multiple groups were conducted using one‐way analysis of variance (ANOVA) followed by Tukey’s post hoc test. Statistical significance was defined as *p*  < 0.05. Detailed numbers of replicates for the analytical specificity and sensitivity assays are specified in the corresponding figure legends. For field sample validation, the 71 pooled tick samples and 53 clinical livestock blood samples were treated as independent biological samples, and each sample was tested once by both the RPA‐CRISPR/Cas12a assay and conventional PCR to assess the sample‐level detection agreement.

## 3. Results

### 3.1. Principle of a One‐Pot RPA‐CRISPR/Cas12a Detection Method for *Babesia* spp. Infecting Cattle and Sheep

**Figure 1:**
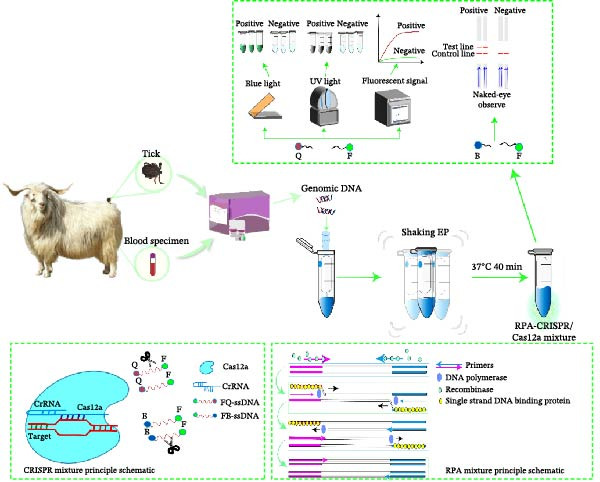
Schematic illustration of the closed‐tube, one‐pot RPA‐CRISPR/Cas12a platform for genus‐level detection of *Babesia* spp. primarily associated with cattle and sheep. Before reaction initiation, the RPA mixture and CRISPR/Cas12a mixture are spatially separated within the same closed reaction tube. After template DNA extracted from tick samples or cattle/sheep blood samples is added, the reaction components are combined by brief centrifugation and incubated at 37°C for 40 min. Results can be interpreted using three readout formats: real‐time fluorescence curve monitoring; endpoint fluorescence detection using FQ‐ssDNA reporters, which can be visually observed under 365 nm UV‐light or 470 nm blue‐light illumination; and lateral flow strip (LFS) detection using FB‐ssDNA reporters. For LFS analysis, the final reaction product is applied to the sample pad of the strip, and results are interpreted within 3 min according to the appearance of the test and control lines. The complete workflow can be completed within ~60 min.

This study established a closed‐tube, one‐pot RPA‐CRISPR/Cas12a detection platform for the genus‐level detection of *Babesia* spp. primarily associated with cattle and sheep (Figure 1). The core feature of this platform is its spatially separated closed‐tube design: the RPA mixture and CRISPR/Cas12a mixture are preloaded in the tube bottom and tube cap, respectively, and combined by a single brief centrifugation step within the fully sealed reaction system. This layout reduces premature contact between amplification and detection reagents before reaction initiation, eliminates the need for postamplification tube opening, and helps reduce the potential risk of amplicon carryover contamination. The assay was performed at a constant 37°C, with the incubation step completed within 40 min. The full workflow, including target amplification, signal generation, and result interpretation, can be finished in ~60 min. The platform supports real‐time fluorescence curve monitoring, endpoint fluorescence visualization under blue‐light or UV excitation using FQ‐ssDNA reporters, and LFS detection using FB‐ssDNA reporters, allowing flexible result judgment under both laboratory‐ and field‐oriented conditions. Overall, this platform provides a rapid, analytically sensitive, and operationally simple approach for field‐oriented genus‐level screening of cattle‐ and sheep‐associated *Babesia* spp.

### 3.2. Establishment of an RPA‐CRISPR/Cas12a Detection System for *Babesia* spp.

**Figure 2:**
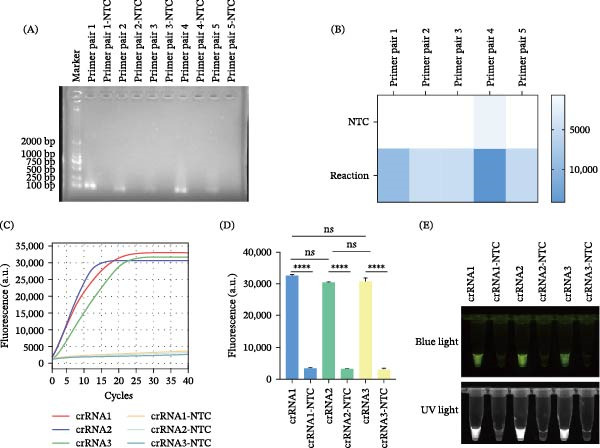
Optimization of RPA primer and crRNA combinations for the detection platform. (A) Selection of five pairs of *Babesia* spp.‐specific RPA primers by analyzing amplification products using 2% agarose gel electrophoresis after incubation at 37°C for 30 min. (B) ImageJ‐based quantitative analysis of band intensities in (A). (C–E) *Babesia* genomic DNA at 157.5 ng/µL was used as the template and added to the RPA‐CRISPR/Cas12a reaction system, followed by incubation at 37°C for 40 min for crRNA screening. Real‐time fluorescence signals were collected (C), endpoint fluorescence intensities were measured (D), and visual fluorescence results under UV or blue‐light illumination were observed (E). Data are presented as mean ± standard deviation (SD). “NTC” represents the no‐template control, and “ns” indicates no statistical significance. Statistical significance was defined as  ^∗∗∗∗^ *p* < 0.0001.

In the RPA‐CRISPR/Cas12a detection system, the selection of primers and crRNAs is crucial for constructing an efficient detection platform. Primers determine the specificity and efficiency of RPA amplification, providing specific templates for subsequent reactions. As the guide component of Cas12a, crRNA directly affects the system’s recognition precision, detection sensitivity, and reliability of the results. Therefore, systematic screening of both primers and crRNAs is essential for achieving high sensitivity and specificity in this assay. In this study, five pairs of RPA candidate primers and three crRNAs were designed based on the conserved gene sequences of the target pathogen. First, RPA amplification products were preliminarily screened by 2% agarose gel electrophoresis (Figure 2A), followed by quantitative analysis (Figure 2B). The results showed that all primer pairs successfully amplified the target bands, with the fourth pair producing the brightest, most distinct, and specific‐looking band, indicating the best amplification performance. Based on these results, three candidate crRNAs were further evaluated for fluorescence signal intensity. Fluorescence signals and endpoint fluorescence data were recorded in the same RPA‐CRISPR/Cas12a reaction system (Figure 2C,D), and images of the final products were taken under UV or blue‐light illumination (Figure 2E). The first crRNA produced the strongest fluorescence signal, demonstrating the highest trans‐cleavage activity among the tested crRNAs. Based on these qualitative and quantitative results, the fourth pair of RPA primers and the first crRNA were identified as the optimal combination for this detection system, laying a reliable experimental foundation for subsequent high‐sensitivity and high‐specificity nucleic acid detection, and were used for subsequent assay development and performance evaluation.

### 3.3. Optimization of the Closed‐Tube, One‐Pot RPA‐CRISPR/Cas12a Detection System

**Figure 3:**
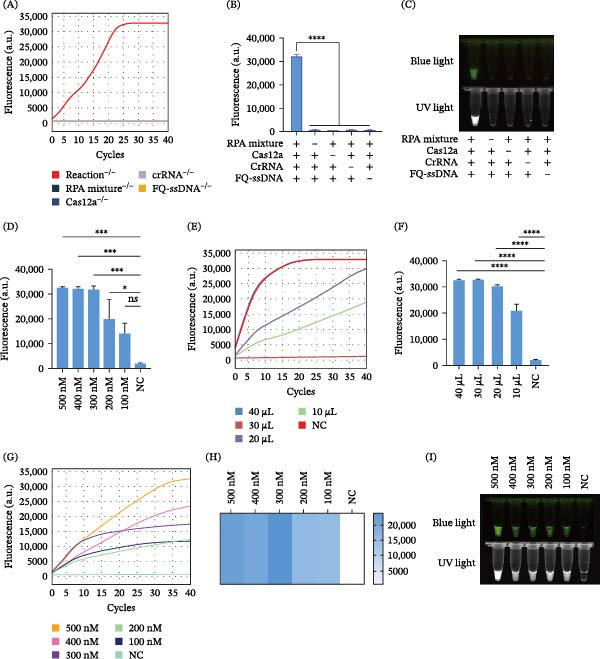
Optimization of the RPA‐CRISPR/Cas12a detection system for *Babesia* spp. (A–C) After incubation at 37°C for 40 min, the complete reaction system containing RPA premix, crRNA, Cas12a, and FQ‐ssDNA, together with corresponding component‐omission control groups, was evaluated by real‐time fluorescence monitoring (A), endpoint fluorescence imaging under UV or blue‐light illumination (B), and endpoint fluorescence intensity measurement (C). (D) Optimization of RNP complex concentration using six concentrations: 500, 400, 300, 200, 100, and 0 nM. (E, F) Optimization of RPA premix volume using 40, 30, 20, 10, and 0 μL, while keeping the CRISPR component volume constant and adjusting the total reaction volume with nuclease‐free water. Real‐time fluorescence signals (E) and endpoint fluorescence intensities (F) were collected after incubation. (G–I) Optimization of FQ‐ssDNA reporter concentration using 500, 400, 300, 200, and 100 nM, with a no‐probe control. Different probe concentrations were analyzed by real‐time fluorescence monitoring (G), endpoint fluorescence heatmap analysis (H), and fluorescence visualization under UV or blue‐light illumination (I). All experiments used 157.5 ng/μL of *Babesia* genomic DNA as the template. “NTC” indicates the no‐template control, and “ns” indicates no statistical significance.  ^∗^ *p* < 0.05;  ^∗∗∗^ *p* < 0.001;  ^∗∗∗∗^ *p* < 0.0001 were considered statistically significant.

To verify the feasibility of the closed‐tube, one‐pot RPA‐CRISPR/Cas12a detection system, a complete reaction mixture containing the RPA premix, crRNA, Cas12a, and reporter molecules was established, together with corresponding component‐omission control groups. After incubation at 37°C for 40 min, only the complete reaction system generated a pronounced fluorescence signal. Both the real‐time fluorescence curves (Figure 3A) and endpoint fluorescence intensities (Figure 3B) were significantly higher than those of all control groups. Consistently, under UV or blue‐light excitation, a clear and visually observable fluorescence signal was detected exclusively in the complete reaction group (Figure 3C), indicating that all four components are essential for effective signal generation.

The RNP complex formed by crRNA and Cas12a serves as the core functional unit of the RPA‐CRISPR/Cas12a system. To determine the optimal RNP working concentration, six concentrations (500, 400, 300, 200, 100, and 0 nM) were evaluated. Endpoint fluorescence analysis showed that 300 nM RNP achieved a strong and stable fluorescence signal while maintaining reaction efficiency and ensuring reagent use and was therefore selected as the optimal concentration for subsequent experiments (Figure 3D).

Next, the reaction volume of the RPA premix was optimized by testing volumes of 40, 30, 20, 10, and 0 μL, while keeping the volume of CRISPR components constant and adjusting the total reaction volume with nuclease‐free water. Both real‐time fluorescence signals and endpoint fluorescence intensities indicated that a reaction containing 20 μL of RPA premix provided strong signal output and good reaction stability (Figure 3E,F). Accordingly, 20 μL was selected as the recommended working volume.

Furthermore, the concentration of the FQ‐ssDNA reporter molecule was optimized by evaluating five concentrations (500, 400, 300, 200, and 100 nM), with a no‐probe reaction serving as a negative control. Among the tested concentrations, 500 nM FQ‐ssDNA produced strong and stable fluorescence signals at early reaction stages, the highest endpoint fluorescence intensity, and clear visual signals under UV or blue‐light excitation (Figure 3G–I). This concentration effectively minimized background interference while ensuring signal stability and was therefore selected for subsequent assays.

### 3.4. Evaluation of the Specificity and Sensitivity of an RPA‐CRISPR/Cas12a Assay for *Babesia*

**Figure 4:**
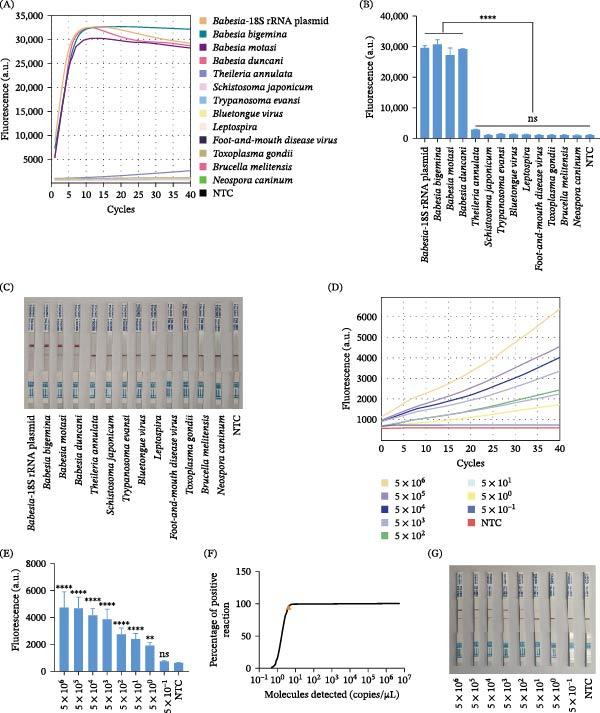
Analytical specificity and sensitivity evaluation of the RPA‐CRISPR/Cas12a detection system. (A–C) The specificity of the established RPA‐CRISPR/Cas12a detection system was evaluated using target *Babesia* templates, including the recombinant *Babesia*‐18S rRNA plasmid, *Babesia bigemina*, *Babesia motasi*, and *Babesia duncani*, together with representative nontarget pathogens. After incubation at 37°C for 40 min under optimized conditions, real‐time fluorescence monitoring (A), endpoint fluorescence intensity analysis (B), and LFS detection (C) were performed. Data in (B) were derived from three independent replicates. (D–G) Sensitivity was evaluated using 10‐fold serial dilutions of the recombinant *Babesia* 18S rRNA plasmid ranging from 5 × 10^6^ to 5 × 10^−1^ copies/μL. Real‐time fluorescence signals (D) and endpoint fluorescence intensities (E) were collected after incubation at 37°C for 40 min. Each concentration in the sensitivity assay shown in (E) was tested in eight independent fluorescence reactions. The detection–probability analysis based on these replicate reactions is shown in (F), with an estimated fluorescence LoD of 5 copies/μL at a 95% detection probability (95% CI: 1.711–81.955 copies/μL). Sensitivity was further validated in parallel using LFS (G). The LFS results were interpreted as positive when both the control line and test line were visible. NTC refers to the no‐template negative control, and all experimental groups were compared with the NTC group. ns, not significant;  ^∗^ *p* < 0.05;  ^∗∗^ *p* < 0.01;  ^∗∗∗^ *p* < 0.001;  ^∗∗∗∗^ *p* < 0.0001 were considered statistically significant. LFS results in the analytical specificity and sensitivity assays were interpreted by personnel blinded to the expected results.

The genus‐level detection range and analytical specificity of the established RPA‐CRISPR/Cas12a assay were evaluated using the *Babesia*‐18S rRNA plasmid, genomic DNA from *Babesia bigemina* (primarily infecting cattle), and *Babesia motasi* (primarily infecting sheep), as well as other pathogens including *Theileria annulata*, *Schistosoma japonicum*, *Trypanosoma evansi*, *bluetongue virus*, *Leptospira*, *foot-and-mouth disease virus*, *Toxoplasma gondii*, *Brucella melitensis*, and *Neospora caninum*. In addition, to further validate the broad‐range detection capability at the genus level, this study included *Babesia duncani*, a species known to infect both mice and humans, in the specificity experiments. Marked fluorescence enhancement was observed exclusively in *Babesia*‐18S rRNA‐positive plasmids, *Babesia bigemina*, *Babesia motasi*, and *Babesia duncani*, whereas no positive signal above the threshold was observed in heterologous pathogen controls or negative controls (Figure 4A,B). Although a weak fluorescence background signal was observed for *Theileria annulata* at a high template concentration of 5 × 10^6^ copies/μL, it remained below the positivity threshold. LFS analysis further verified the specificity of the assay: specific test bands appeared only in *Babesia*‐18S rRNA‐positive plasmids, *Babesia bigemina*, *Babesia motasi*, and *Babesia duncani* (Figure 4C), indicating no LFS‐detectable cross‐reactivity under the tested conditions. In addition, ImageJ‐based quantitative analysis of the LFS test line intensity further supported the visual interpretation, showing that clear band signals were detected only in the *Babesia*‐positive groups, whereas the heterologous pathogen controls and negative controls remained at background levels (Figure S4). These findings demonstrate that the developed RPA‐CRISPR/Cas12a assay possesses high specificity and reliable broad‐range detection capability for *Babesia* at the genus level. Nevertheless, the RPA‐CRISPR/Cas12a readout itself does not provide species‐level discrimination, and further sequencing or species‐specific assays are required when precise species confirmation is needed.

To assess the analytical sensitivity of the assay using plasmid standards, a recombinant plasmid of *Babesia*‐18S rRNA was serially diluted 10‐fold, ranging from 5 × 10^6^ to 5 × 10^−1^ copies/μL. Real‐time fluorescence monitoring (Figure 4D), endpoint fluorescence measurements (Figure 4E), and LFS validation (Figure 4G) were performed. Each concentration was evaluated in eight independent fluorescence reactions. The proportions of positive reactions at each concentration were used to construct a detection–probability curve (Figure 4F). Based on this replicate‐supported analysis, the fluorescence‐based assay achieved an estimated LoD of 5 copies/μL at a 95% detection probability (95% CI: 1.711–81.955 copies/μL) under the tested conditions. Endpoint fluorescence intensity was positively correlated with the template concentration. The visual detection limit of the LFS assay for plasmid templates was 50 copies/μL. In addition, ImageJ‐based quantitative analysis of the LFS test‐line intensity further supported the visual LFS results, showing a concentration‐dependent decrease in band intensity and confirming 50 copies/μL as the detection limit of the LFS assay under the tested conditions (Figure S5). For the analytical specificity and analytical sensitivity assays, the LFS results were visually interpreted by personnel blinded to the expected sample identities and template concentrations. For comparison, the analytical sensitivity of conventional PCR was also evaluated using 10‐fold serially diluted PCR‐specific plasmid standards. As shown in Figure S6, the expected 452‐bp specific band was observed from 5 × 10^2^ to 5 × 10^6^ copies/μL, whereas no specific band was detected in the negative control or at lower template concentrations. Therefore, the detection limit of conventional PCR was determined to be 500 copies/μL under the tested conditions.

### 3.5. Evaluation of the Established RPA‐CRISPR/Cas12a Assay in a Plasmid‐Spiked Blood Matrix

**Figure 5:**
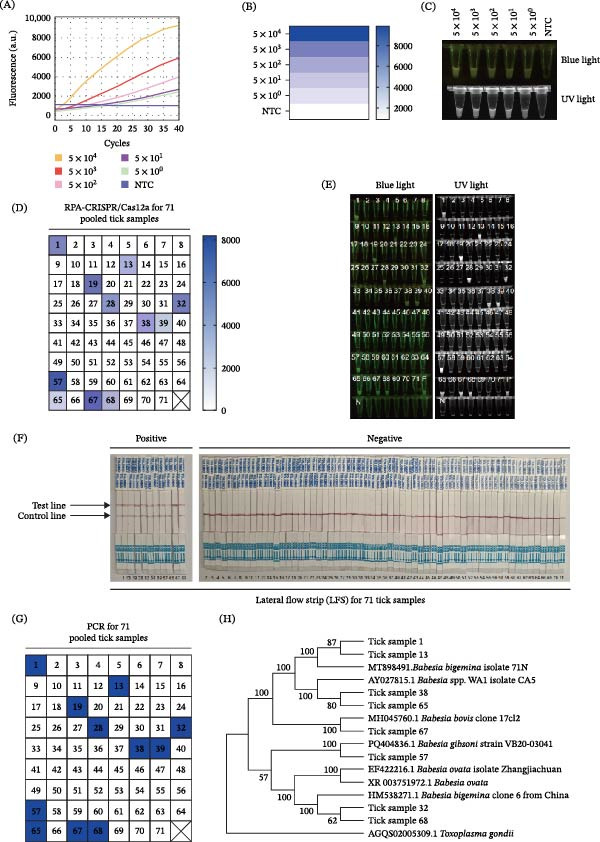
Validation of the RPA‐CRISPR/Cas12a assay using pooled tick samples and plasmid‐spiked blood samples. (A–C) Detection results of plasmid‐spiked blood samples using the RPA‐CRISPR/Cas12a assay. Following incubation under optimized conditions, real‐time fluorescence signals (A), heatmap representation of fluorescence intensities (B), and visual detection results under blue‐light and UV illumination (C) were obtained for the plasmid‐spiked blood samples. (D) Heatmap analysis of endpoint fluorescence signals obtained from genomic DNA extracted from 71 pooled tick samples using the RPA‐CRISPR/Cas12a assay. Color intensity corresponds to fluorescence signal strength, with darker colors indicating positive results and lighter colors indicating negative results. (E) Endpoint fluorescence images of the 71 pooled tick samples captured under blue‐light and UV excitation after completion of the RPA‐CRISPR/Cas12a reaction. The visual results are fully consistent with the heatmap analysis. (F) Detection results of the 71 pooled tick samples using LFS based on the RPA‐CRISPR/Cas12a assay. Samples showing two distinct bands were interpreted as positive, while those showing only a control band were interpreted as negative. (G) Conventional PCR results for the 71 pooled tick samples analyzed using ImageJ software. Dark‐colored cells indicate positive results, whereas white cells indicate negative results. (H) Phylogenetic analysis of PCR‐positive tick‐pool amplicons after Sanger sequencing of all 11 positive tick pools. Identical or nearly identical sequences were represented by one sequence to avoid redundancy, with tick sample 1 selected as the representative sequence for tick samples 1, 19, 28, and 39. The phylogenetic tree was constructed using the neighbor‐joining (NJ) method, and reference sequences of different *Babesia* spp. were retrieved from the GenBank database.

Because of the complexity of biological sample matrices, it is necessary to evaluate whether blood components interfere with the assay performance. Considering that *Babesia* spp. primarily parasitize erythrocytes, the RPA‐CRISPR/Cas12a assay was further evaluated using plasmid‐spiked blood samples as a simulated blood matrix model. The assay exhibited stable signal generation during real‐time monitoring (Figure 5A), suggesting that the spiked blood matrix did not markedly reduce detectable assay signals under the tested conditions. Endpoint fluorescence analysis (Figure 5B) further confirmed a consistent performance across samples. Notably, the detection outcomes remained readily distinguishable through direct visual inspection under blue‐light and UV illumination (Figure 5C), indicating the potential suitability of this platform for rapid visual detection. Consistent and distinguishable positive signals were obtained from blood samples spiked with recombinant plasmid templates at concentrations down to 5 copies/μL, whereas negative control samples remained negative. These results indicate that the assay maintained detectable performance in a plasmid‐spiked blood matrix under the tested conditions.

### 3.6. The Practical Validation of the RPA‐CRISPR/Cas12a‐Based Diagnostic Platform

To evaluate the field applicability and genus‐level detection performance of the RPA‐CRISPR/Cas12a assay, a total of 71 pooled tick samples and 53 clinical blood samples from cattle and sheep were analyzed. Genomic DNA was extracted from all samples and used as templates for parallel analysis using the RPA‐CRISPR/Cas12a assay and conventional PCR. Endpoint fluorescence heatmap analysis revealed that 11 tick sample pools exhibited fluorescence signals higher than the positivity threshold, while the remaining 60 samples showed signals at or below the threshold (Figure 5D). Visual inspection under UV and blue‐light excitation yielded results fully consistent with the fluorescence data (Figure 5E). Combined with LFS analysis, the overall *Babesia* positivity rate in pooled tick samples was determined to be 15.49% (11/71) (Figure 5F). Then, PCR analysis identified the same 11 samples as positive (Figure S7), corresponding to a positivity rate of 15.49% (11/71) (Figure 5G). For the 53 clinical livestock blood samples, endpoint fluorescence heatmap analysis identified five positive samples with obvious fluorescence signals, whereas the remaining 48 samples were negative (Figure S8A). The results obtained by blue and UV‐light visualization (Figure S8B) and LFS detection were consistent with the endpoint fluorescence results (Figure S8E), giving an overall *Babesia* spp. positivity rate of 9.43% (5/53). Conventional PCR detected the same five positive samples (Figure S9), showing 100% concordance with the RPA‐CRISPR/Cas12a assay (Figure S8C). Further analysis using 2 × 2 contingency tables showed 100.0% sensitivity, specificity, positive and negative predictive values, and overall agreement relative to conventional PCR for both sample sets. The Cohen’s kappa value was 1.000, indicating excellent consistency between the two methods (Tables S3 and S4).

To further confirm that the positive amplicons belonged to *Babesia* spp. and to provide phylogenetic information, all 11 PCR‐positive tick pools and all five PCR‐positive clinical blood samples were subjected to Sanger sequencing. To avoid redundancy in the phylogenetic tree, identical or nearly identical sequences were represented by a single representative sequence. Specifically, tick sample 1, tick sample 19, tick sample 28, and tick sample 39 showed identical sequences and were represented by Tick sample 1 in the phylogenetic tree. Phylogenetic analysis based on the neighbor‐joining method showed that sequences from Shanxi tick samples clustered with *Babesia bigemina* and *Babesia* sp. WA1 isolate CA5, while Jilin tick samples clustered with *Babesia bigemina*, *Babesia bovis*, *Babesia gibsoni*, and *Babesia* sp. *WA1 isolate CA5* (Figure 5H). For the clinical blood samples, four positive sequences clustered with *Babesia ovis*, whereas one sequence clustered with *Babesia crassa* (Figure S8D). These results further supported the reliability of the RPA‐CRISPR/Cas12a assay for the genus‐level detection of *Babesia* spp. in pooled tick and clinical livestock blood samples.

## 4. Discussion

Babesiosis, a significant tick‐borne zoonotic parasitic disease, continues to pose a persistent threat to both public health and the livestock industry worldwide [1]. In China, it is classified as a Category III animal epidemic, and infected animals typically exhibit symptoms such as high fever, anemia, emaciation, and loss of appetite [39]. If left untreated, the mortality rate can rise to 30%­–50% [40]. Traditional diagnostic methods primarily rely on microscopic examination [41], which, despite being simple and easy to perform, has its sensitivity and accuracy heavily dependent on the microscopist’s expertise. Moreover, it is prone to false negatives, especially in earlystage infections or samples with low parasitemia, making it inadequate for large‐scale screening or field monitoring [42]. Although molecular diagnostic techniques, such as PCR, offer high sensitivity and specificity, their reliance on specialized equipment, stable power supply, and skilled personnel significantly limits their applicability in resource‐limited settings, primary care clinics, and field conditions [43]. Therefore, the development of an on‐site rapid, cost‐effective, and easy‐to‐use diagnostic tool with reliable analytical performance is crucial for the early detection, epidemiological surveillance, and effective control of babesiosis.

This study successfully developed an integrated and visual genus‐level detection platform for *Babesia* spp. based on RPA‐CRISPR/Cas12a. The platform enables RPA amplification and Cas12a‐mediated detection to occur in a single closed reaction tube through a one‐pot strategy. Although Cas12a‐mediated signal generation occurs following target recognition and increases as RPA amplicons accumulate, the two processes temporally overlap during incubation rather than being manually performed as two sequential steps. The one‐pot format was adopted to simplify the workflow, avoid postamplification tube opening, and help reduce the potential risk of carryover contamination, thereby supporting the potential use of the assay in POC and field‐oriented applications. RPA allows rapid exponential amplification of target nucleic acids under constant low‐temperature conditions [24], effectively overcoming the insufficient signal intensity of CRISPR‐based detection systems when dealing with trace‐level templates [44].

In fully integrated one‐pot systems, premature activation of Cas12a may lead to the cleavage of primers and newly synthesized amplicons, thereby reducing amplification efficiency [35]. To reduce the potential interference of premature Cas12a activation with RPA amplification, the assay was designed as a closed‐tube, one‐pot format. The RPA mixture and CRISPR/Cas12a mixture were spatially separated before reaction initiation and then combined by brief centrifugation within the closed tube. This design reduces premature contact between Cas12a components and the RPA mixture while maintaining the advantages of simplified operation and reduced carryover contamination risk. To assess whether the incorporation of Cas12a detection components affected RPA amplification, RPA reactions containing Cas12a detection components were compared with RPA‐only reactions by agarose gel electrophoresis and ImageJ‐based band‐intensity analysis. Clear amplification bands were observed in all three independent positive reactions in both groups, whereas the corresponding no‐template controls remained negative (Figure S10A). Furthermore, the band intensities of both positive reaction groups were significantly higher than those of their corresponding no‐template controls, while no significant difference was observed between the RPA with Cas12a group and the RPA reaction (Figure S10B). These results indicate that, under the optimized reaction conditions used in this study, the incorporation of the Cas12a detection components did not significantly impair endpoint RPA amplification. After incubation at 37°C for 40 min, the detection results could be directly obtained and interpreted. Upon specific recognition of the dsDNA target, the Cas12a–crRNA complex activates its trans‐cleavage activity, which nonspecifically cleaves ssDNA reporters [33]. This process enables efficient conversion of nucleic acid signals into three visual readouts: fluorescence signals detectable using portable UV or blue‐light devices (365 or 470 nm) or visible band signals that can be directly interpreted by the naked eye using LFS.

Although several RPA‐CRISPR/Cas12a diagnostic systems have been reported, most rely on a single readout modality. For example, RPA‐CRISPR/Cas12a assays for porcine reproductive and respiratory syndrome virus [45] and human bocavirus 1 [46] primarily visualize results via fluorescence under UV and blue light. Other systems such as those developed for universal dengue virus detection [47] or deformed wing virus [48] rely chiefly on lateral flow‐based readout. In parasite detection, an ERA‐CRISPR/Cas12a assay has recently been developed for bovine *Theileria annulata*, enabling visual interpretation under UV and blue light and achieving a detection limit of 10 copies/μL within 40 min [49]. In contrast, our platform supports three visual readout formats, including blue‐light fluorescence visualization, UV fluorescence visualization, and LFS detection. In addition, fluorescence signals can be directly measured using a fluorescence reader or a qPCR instrument. This flexibility broadens its potential applicability across diverse scenarios, from laboratories to POCT in resource‐limited settings. From a workflow perspective, representative two‐step RPA‐CRISPR/Cas12a assays generally require postamplification transfer of RPA products into a separate Cas12a detection mixture. As summarized in Table S5, the present platform avoids this transfer step through a closed‐tube, one‐pot configuration and provides real‐time fluorescence monitoring, endpoint fluorescence visualization, and LFS‐based interpretation. However, because the target genes, template types, and experimental conditions differ among studies, these comparisons provide only descriptive methodological context and should not be regarded as direct evidence of superior analytical performance over two‐step assays. Overall, the closed‐tube RPA‐CRISPR/Cas12a assay developed in this study provides a rapid and operationally simple approach for the genus‐level detection of *Babesia* spp. The closed‐tube reaction format reduces the risk of carryover contamination, thereby supporting simplified operation and reliable endpoint interpretation.

*Babesia* spp. are widely prevalent globally. To validate the assay’s field applicability and reliability for genus‐level detection, we tested 71 pooled tick samples and observed 100% concordance with PCR in testing 71 tick samples, detecting 11 positive samples (7 from Shanxi Province and 4 from Jilin Province; positivity rate: 15.49%). Since the tick samples in this study were analyzed in pooled form, the positive results are best interpreted as evidence of the presence and circulation of *Babesia* spp. in the local tick population, providing useful information for vector‐based surveillance and potential transmission‐risk assessment, but they do not directly reflect the infection status of livestock hosts. To further evaluate the applicability of the assay in host‐derived samples, 53 clinical blood samples from cattle and sheep collected in Shanxi Province were tested. The assay also showed 100% concordance with conventional PCR and identified five positive samples, corresponding to a positivity rate of 9.43%. Sanger sequencing and phylogenetic analysis of PCR‐positive samples confirmed that the positive samples belonged to *Babesia* spp. and provided phylogenetic reference information for the detection results. These results also emphasized that the RPA‐CRISPR/Cas12a assay developed in this study is a broad‐range genus‐level detection tool without an inherent species discrimination capacity. Positive samples requiring precise species identification should therefore be further analyzed by sequencing or species‐specific assays. The assay showed stable detection performance in both field‐collected pooled tick samples and clinical livestock blood samples, verifying its applicability for genus‐level screening. It can support both vector surveillance and host infection screening and may be particularly useful for epidemiological screening and field monitoring in regions with coendemic *Babesia* species, offering practical value for the integrated prevention and control of babesiosis.

This complementary validation strategy using two sample types not only strengthens the practical application potential of the assay but also enables a more comprehensive and balanced interpretation of detection findings at both the vector and host levels. As *Babesia* spp. primarily parasitize erythrocytes and can be transmitted via blood [50], plasmid‐spiked blood samples were further used to evaluate the applicability of the assay in a blood matrix. The RPA‐CRISPR/Cas12a assay achieved a fluorescence detection limit of 5 copies/μL for both recombinant plasmid templates and plasmid‐spiked blood samples, indicating that the blood matrix did not markedly reduce analytical sensitivity in this spiked‐sample model. However, this value should be interpreted as an analytical detection limit based on recombinant plasmids or plasmid‐spiked blood samples rather than a detection limit determined from naturally infected blood samples. As the reference method, conventional PCR was also evaluated in this study, showing a detection limit of 500 copies/μL and requiring ~1.5–2 h for the complete detection workflow. In comparison, the RPA‐CRISPR/Cas12a assay achieved a fluorescence detection limit of 5 copies/μL and an LFS detection limit of 50 copies/μL within 40 min at 37°C, suggesting better analytical sensitivity and a shorter detection time under the tested conditions. qPCR‐based methods, including qPCR [51], qPCR‐HRM [52], and Pan‐*Babesia* FRET‐qPCR [53], generally achieve high analytical sensitivity but depend on fluorescence detection instruments. Isothermal amplification methods such as LAMP [54] and RPA‐LFD [55] shorten the detection time to ~30–60 min, but their detection spectrum or readout format is relatively limited (Table S6). Overall, the platform developed in this study provides a rapid, sensitive, and flexible approach for the genus‐level detection of *Babesia* spp. It is noteworthy that the detection sensitivity may vary among different pathogens and sample matrices. For instance, LAMP detection of *Brucella* can be matrix‐dependent in blood samples [56], while qPCR detection of *Leishmania* may show variable performance across blood samples [57], and molecular assays for *Leptospira* in whole blood may exhibit reduced sensitivity [58]. These variations further emphasize the need for matrix‐specific validation when applying molecular assays to complex biological samples.

Notably, a weak fluorescence signal was observed for *Theileria annulata* at a very high template concentration of 5 × 10^6^ copies/μL, although it remained below the positivity threshold established for the assay. We interpret this as a low‐level background signal rather than true cross‐reactivity, attributable to three combined factors. First, *Babesia* and *Theileria* are both members of *Piroplasmida* and share partial sequence conservation in the 18S rRNA gene; as a broad‐range genus‐level assay targeting the conserved 18S rRNA locus, low‐level background reactivity at very high template concentrations may occur in closely related piroplasmids and should therefore be interpreted cautiously [59]. Second, RPA relies on recombinase‐mediated primer targeting and strand‐displacement DNA synthesis, and primer‐template mismatches can influence RPA specificity in a position‐dependent manner; therefore, highly homologous nontarget templates at very high copy numbers may support inefficient amplification [60]. Third, Cas12a retains partial trans‐cleavage activity in the presence of minor mismatches between crRNA and the protospacer, which can further amplify weak signals from inefficiently amplified nontarget amplicons.

Importantly, this weak background signal was only detectable via the fluorescence readout and was completely absent in the LFS assay. At the same high template concentration, only *Babesia* spp. produced clear test lines on the strips, while *Theileria annulata* and all other nontarget pathogens yielded negative results. This discrepancy indicates that the weak fluorescence does not represent equivalent positive reactivity and that the LFS readout showed no detectable cross‐reactivity under the tested conditions.

This observation is consistent with the general practice in the field. Broad‐range 18S rRNA‐based molecular assays for piroplasmids are widely recognized as initial screening tools rather than definitive species‐level identification methods, and positive results usually require confirmation by sequencing, RFLP, or species‐specific PCR [59]. The classical reverse line blot assay similarly employs universal 18S rRNA amplification for *Theileria* and *Babesia*, followed by species‐specific probes for discrimination, further demonstrating that broad 18S amplification does not equate to definitive taxonomic identification [60]. Accordingly, we propose that in practical application, positive or suspicious results, especially fluorescence‐only weak signals, should be further confirmed by sequencing or species‐specific assays. In regions where *Theileria* and *Babesia* are coendemic, further confirmation via sequencing or alternative targets such as cytb, cox1, or ITS is recommended for positive or suspicious samples [61].

However, several limitations of this method should be acknowledged. First, field implementation is currently limited by the reliance on nucleic acid extraction and cold‐chain storage to maintain Cas12a and crRNA stability, as well as the need for portable detection devices. To achieve fully equipment‐independent on‐site diagnostics, the future integration of extraction‐free protocols and lyophilized reagents is essential. Second, although agarose gel electrophoresis and Image J‐based band‐intensity analysis indicated that the incorporation of Cas12a detection components did not significantly impair endpoint RPA amplification under the optimized conditions used in this study, a direct comparison between the present closed‐tube, one‐pot system and a conventional two‐step RPA‐CRISPR/Cas12a assay was not performed under matched experimental conditions. Therefore, the advantages of the one‐pot format should primarily be interpreted as workflow‐related advantages, including reduced handling steps, avoidance of postamplification tube opening, and a lower potential risk of amplicon carryover contamination, rather than experimentally demonstrated superiority in analytical performance over two‐step assays. Future studies should conduct direct comparisons using the same target, sample matrix, and reaction conditions to further evaluate the differences between the two formats in amplification efficiency, analytical performance, reproducibility, and operational convenience. Third, the current assay does not incorporate an internal amplification control (IAC) to monitor reaction failure, inadequate DNA extraction, or the presence of amplification inhibitors in complex field samples. The nonspecific trans‐cleavage activity of Cas12a presents an inherent technical challenge for integrating a conventional IAC into the closed‐tube, one‐pot system: once activated by target amplicons, Cas12a would indiscriminately cleave the reporter probe of the IAC, making it difficult to distinguish target and IAC signals in a simple single‐reporter reaction. Accordingly, negative results should be interpreted with caution, particularly for field‐collected samples that may contain inhibitory substances. Future optimization will focus on developing compatible internal control strategies, such as parallel‐reaction IACs or spatially separated dual‐chamber systems, to further enhance the reliability of on‐site diagnosis. Fourth, the current platform is designed for the genus‐level detection of *Babesia* spp. and does not support species‐level discrimination or multiplex detection, limiting its application in mixed infections or complex samples. Therefore, species‐level confirmation still requires sequencing or other species‐specific assays. Fifth, although the LFS results in the analytical specificity and sensitivity assays were visually interpreted by personnel blinded to the expected results, the comparative validation using pooled tick samples and clinical livestock blood samples was not conducted in a fully blinded manner. In addition, conventional PCR was used as the comparator method rather than an independent gold standard. Therefore, the current performance estimates should be interpreted as preliminary and require further validation using larger sample sets and fully blinded study designs. Future research will focus on simplified nucleic acid preparation and multiplex detection strategies based on multi‐target crRNAs or multi‐Cas enzymes to further enhance the versatility and applicability of the platform in field settings.

In conclusion, the RPA‐CRISPR/Cas12a integrated detection platform established in this study combines the efficiency of isothermal amplification with the sequence‐recognition capability of the CRISPR system, providing a rapid, analytically sensitive, and specific method for the genus‐level detection of *Babesia* spp. with reduced dependence on complex instrumentation. The closed‐tube format reduces the risk of carryover contamination, and the three visual readout options improve the flexibility of result interpretation under different testing conditions. The platform demonstrated reliable performance, with results fully concordant with those of conventional PCR in the validation of both pooled tick samples and clinical blood samples. Although pooled tick samples mainly support its application in vector‐based surveillance, the additional validation using clinical blood samples from cattle and sheep further extends its potential value for host infection screening. Therefore, this platform may serve as a useful tool for rapid on‐site detection, preliminary surveillance, and field‐based monitoring of babesiosis, thereby supporting disease control, public health protection, and livestock health management. Nevertheless, larger‐scale validation using naturally infected clinical samples will be helpful to further confirm its diagnostic applicability, and species‐level confirmation should still rely on sequencing or other species‐specific assays when precise species identification is required. Future work will focus on further improving field applicability by integrating this platform into portable microfluidic devices and developing lyophilized reagents suitable for room‐temperature transportation and storage, thereby facilitating their use in POCT and resource‐limited settings.

## 5. Conclusion

In conclusion, we have developed a rapid and sensitive closed‐tube, one‐pot RPA‐CRISPR/Cas12a platform for the detection of *Babesia* spp. primarily associated with cattle and sheep. Using recombinant plasmid standards, the fluorescence‐based assay achieved a detection limit of 5 copies/μL, whereas the LFS visual readout showed a detection limit of 50 copies/μL using the same plasmid standards. These results demonstrate the analytical performance of the assay under the experimental conditions. The assay can be completed within 60 min at a constant temperature of 37°C. Results are directly visible through blue‐light or UV illumination or can be interpreted using LFS, while the closed‐tube format avoids postamplification tube opening and helps reduce the potential risk of amplicon carryover contamination. In sample validation, pooled tick samples mainly support the potential use of this platform for vector‐based surveillance, whereas clinical blood samples from cattle and sheep provide preliminary evidence for its application in host infection screening. Given its simplicity, speed, and low equipment requirements, this platform shows potential for on‐site detection in resource‐limited settings, particularly once combined with portable microfluidic devices, lyophilized reagents, and simplified extraction protocols. Further validation using larger numbers of naturally infected clinical samples will be needed to confirm its diagnostic applicability. The assay can also be adapted to detect other pathogens by redesigning the RPA primers and crRNAs. Furthermore, the potential for multiplex detection using orthogonal Cas proteins offers promise for the simultaneous detection of *Babesia* spp. and other related pathogens in future applications.

## Author Contributions

**Wenxue Lu:** conceptualization, writing – original draft. **Qiankun Yang:** formal analysis, methodology. **Peng Zhao:** resources. **Yutong Cao:** data curation. **Zeqian Jing:** software. **Nan Zhang and Xiaocen Wang:** supervision. **Jianhua Li:** validation. **Xin Li and Xu Zhang:** visualization. **Lili Cao:** writing – review and editing. **Pengtao Gong:** project administration, writing – review and editing, investigation.

## Funding

This study was financially supported by the National Key Research and Development Program of China (Grant 2024YFD1800100) and the China Wool‐sheep & Cashmere‐goat Research System (Grant CARS‐39).

## Disclosure

All AI‐assisted suggestions were critically reviewed and approved by the authors to ensure scientific accuracy and integrity.

## Ethics Statement

All animal experiments were approved by the Animal Ethical Committee of Jilin University (Permit Number SY202407006).

## Conflicts of Interest

The authors declare no conflicts of interest.

## Supporting Information

Additional supporting information can be found online in the Supporting Information section.

## Supporting information


**Supporting Information** Figure S1: Expression, purification, and functional validation of His‐LbCas12a. Table S1: The primer sequences for RPA assay. Table S2: The primers used in this study. Figure S2: The concentration and purity of purified crRNAs. Figure S3: Multiple sequence alignment of the 18S rRNA gene from *Babesia* species infecting cattle and sheep. Figure S4: Quantitative signal intensity analysis of lateral flow strip results from the specificity assay. Figure S5: Quantitative signal intensity analysis of lateral flow strip results for the sensitivity assay. Figure S6: Analytical sensitivity evaluation of conventional PCR for the detection of *Babesia* spp. Figure S7: PCR and agarose gel electrophoresis were performed on 71 tick sample pools, including pools 1–41 from Shanxi Province and pools 42–71 from Jilin Province. Figure S8: Detection and phylogenetic confirmation of *Babesia* spp. in clinical livestock blood samples using the RPA‐CRISPR/Cas12a assay. Table S3: Detection results and diagnostic performance of the assay for 71 pooled tick samples. Table S4: Detection results and diagnostic performance of the assay for 53 clinical livestock blood samples. Figure S9: PCR and agarose gel electrophoresis were performed on 53 clinical livestock blood samples. Figure S10: Assessment of the effect of Cas12a detection components on RPA amplification. Table S5: Descriptive comparison of the present closed‐tube, one‐pot RPA‐CRISPR/Cas12a platform with representative two‐step RPA‐CRISPR/Cas12a assays. Table S6: Comparison of the established RPA/Cas12a with other molecular detection methods.

## Data Availability

The sequence data supporting the findings of this study are openly available in the GenBank database under Accession Numbers PZ539632–PZ539642 and PZ528466–PZ528470. Other data supporting the findings of this study are available within the article and from the corresponding author upon reasonable request.
