# Supplementary material for: A One‐Pot RPA‐CRISPR/Cas12a Assay for Rapid Genus‐Level Detection of Babesia spp. in Ticks and Livestock Blood Samples
Source: Transbound Emerg Dis. 2026 Jul 29;2026:9289663. doi: 10.1155/tbed/9289663 (PMC13417015; doi:10.1155/tbed/9289663)
Supplement: Supplementary file 1 — Supporting Information Figure S1: Expression, purification, and functional validation of His‐LbCas12a. Table S1: The primer sequences for RPA assay. Table S2: The primers used in this study. Figure S2: The concentration and purity of purified crRNAs. Figure S3: Multiple sequence alignment of the 18S rRNA gene from Babesia species infecting cattle and sheep. Figure S4: Quantitative signal intensity analysis of lateral flow strip results from the specificity assay. Figure S5: Quantitative signal intensity analysis of lateral flow strip results for the sensitivity assay. Figure S6: Analytical sensitivity evaluation of conventional PCR for the detection of Babesia spp. Figure S7: PCR and agarose gel electrophoresis were performed on 71 tick sample pools, including pools 1–41 from Shanxi Province and pools 42–71 from Jilin Province. Figure S8: Detection and phylogenetic confirmation of Babesia spp. in clinical livestock blood samples using the RPA‐CRISPR/Cas12a assay. Table S3: Detection results and diagnostic performance of the assay for 71 pooled tick samples. Table S4: Detection results and diagnostic performance of the assay for 53 clinical livestock blood samples. Figure S9: PCR and agarose gel electrophoresis were performed on 53 clinical livestock blood samples. Figure S10: Assessment of the effect of Cas12a detection components on RPA amplification. Table S5: Descriptive comparison of the present closed‐tube, one‐pot RPA‐CRISPR/Cas12a platform with representative two‐step RPA‐CRISPR/Cas12a assays. Table S6: Comparison of the established RPA/Cas12a with other molecular detection methods. [file TBED-2026-9289663-s001.docx]

1. **Supplementary Materials**


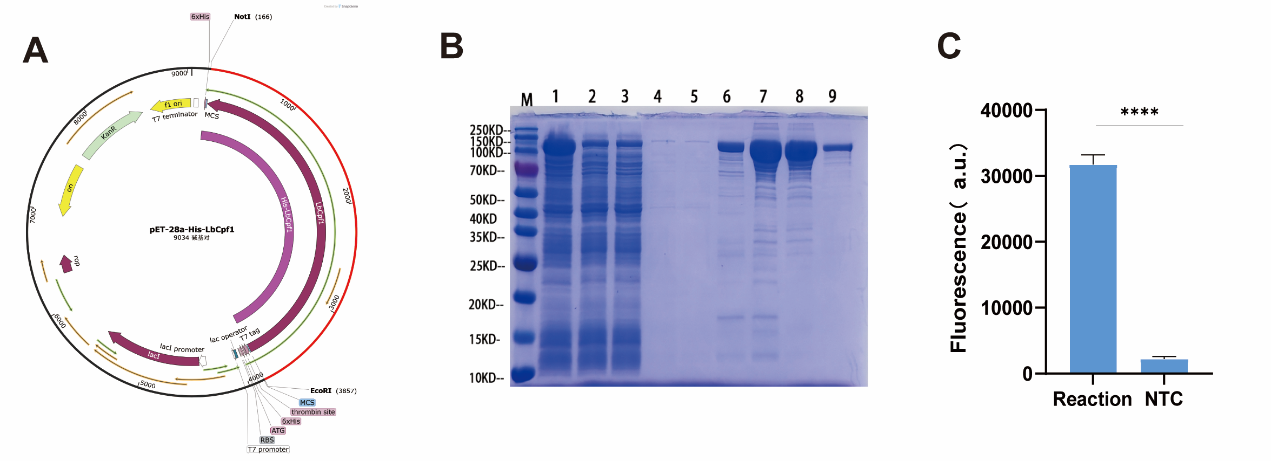


F_IGURE_ S1: Expression, purification, and functional validation of His-LbCas12a. (A) Schematic map of the pET-28a-His-LbCpf1 plasmid. (B) SDS-PAGE analysis of His-LbCas12a purification. M, protein molecular weight marker; lane 1, bacterial lysate after sonication; lanes 2–3, column flow-through fractions; lanes 4–5, column-filtered bacterial solution; lanes 6–9, fractions eluted with increasing concentrations of imidazole (200, 400, 800 nM, and 2.4 M). (C) Trans-cleavage activity assay of recombinant Cas12a. The reaction was incubated at 37 °C for 40 min and contained 250 nM Cas12a, 250 nM crRNA, 200 nM fluorophore-quencher single-stranded DNA reporter, 400 nM target plasmid, and 1× NE Buffer r3.1, with nuclease-free water added to the final volume. NTC indicates the no-template control. Endpoint fluorescence signals were recorded and analyzed.

T_ABLE_ S1: The primer sequences for RPA assay.

| Name | Sequence (5´-3´) | Product(bp) | Position(bp) |
| --- | --- | --- | --- |
| B-F1 | GGAGCCTGAGAAACGGCTACCACATCTA | 101 | 81-109 |
| B-R1 | GCCCTGTATTGTTATTTCTTGTCACTACCTC |  | 145-176 |
| B-F2 | GGAGCCTGAGAAACGGCTACCACATCTA | 101 | 81-109 |
| B-R2 | CCCTGTATTGTTATTTCTTGTCACTACCTCCCT |  | 142-176 |
| B-F3 | ATCAGCTTGACGGTAGGGTATTGGCCTA | 109 | 1-28 |
| B-R3 | CTTCCTTAGATGTGGTAGCCGTTTCTCAG |  | 81-109 |
| B-F4 | ATCAGCTTGACGGTAGGGTATTGGC | 117 | 1-25 |
| B-R4 | CCTGCTGCCTTCCTTAGATGTGGTA |  | 93-117 |
| B-F5 | ATCAGCTTGACGGTAGGGTATTGGC | 111 | 1-25 |
| B-R5 | GCCTTCCTTAGATGTGGTAGCCGTT |  | 87-111 |

T_ABLE_ S2: The primers used in this study.

| Name | Sequence (5´-3´) |
| --- | --- |
| crRNA-F1 | GAAATTAATACGACTCACTATAGGGTAATTTCTACTAAGTGTAGATCGGAGAGGGAGCCTGAGAAA |
| crRNA-R1 | TTTCTCAGGCTCCCTCTCCGATCTACACTTAGTAGAAATTACCCTATAGTGAGTCGTATTAATTTC |
| crRNA-F2 | GAAATTAATACGACTCACTATAGGGTAATTTCTACTAAGTGTAGATGGGTTCGATTCCGGAGAGGG |
| crRNA-R2 | CCCTCTCCGGAATCGAACCCATCTACACTTAGTAGAAATTACCCTATAGTGAGTCGTATTAATTTC |
| crRNA-F3 | GAAATTAATACGACTCACTATAGGGTAATTTCTACTAAGTGTAGATTCAGGCTCCCTCTCCGGAAT |
| crRNA-R3 | ATTCCGGAGAGGGAGCCTGAATCTACACTTAGTAGAAATTACCCTATAGTGAGTCGTATTAATTTC |
| *Babesia*-F | ATCAGCTTGACGGTAGGGTATTG |
| *Babesia*-R | GCCCTGTATTGTTATTTCTTGTC |
| PCR-B-F | GTCTTGTAATTG GAATGATGG |
| PCR-B -R | TAGTTTATGGTTAGGACTACG |


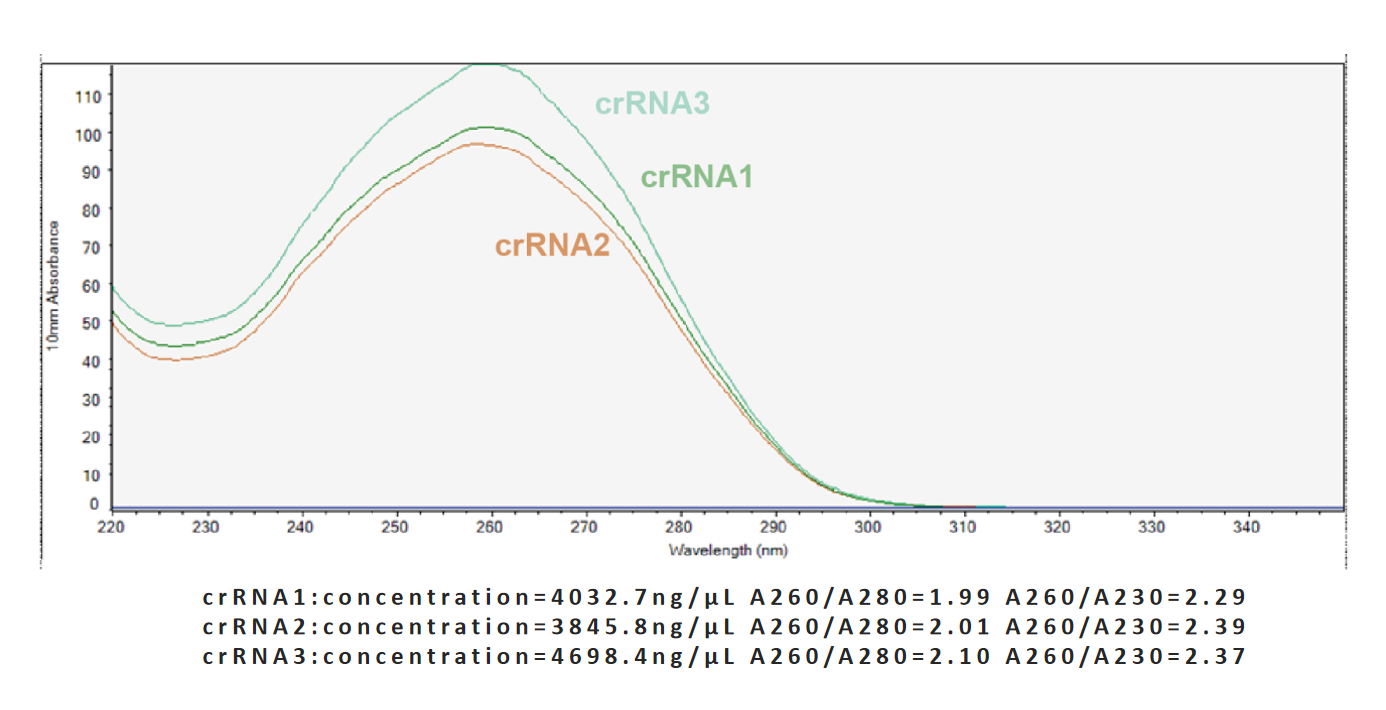


F_IGURE_ S2: The concentration and purity of purified crRNAs. The concentration and purity purified crRNAs of crRNA1, crRNA2 and crRNA3 were measured.


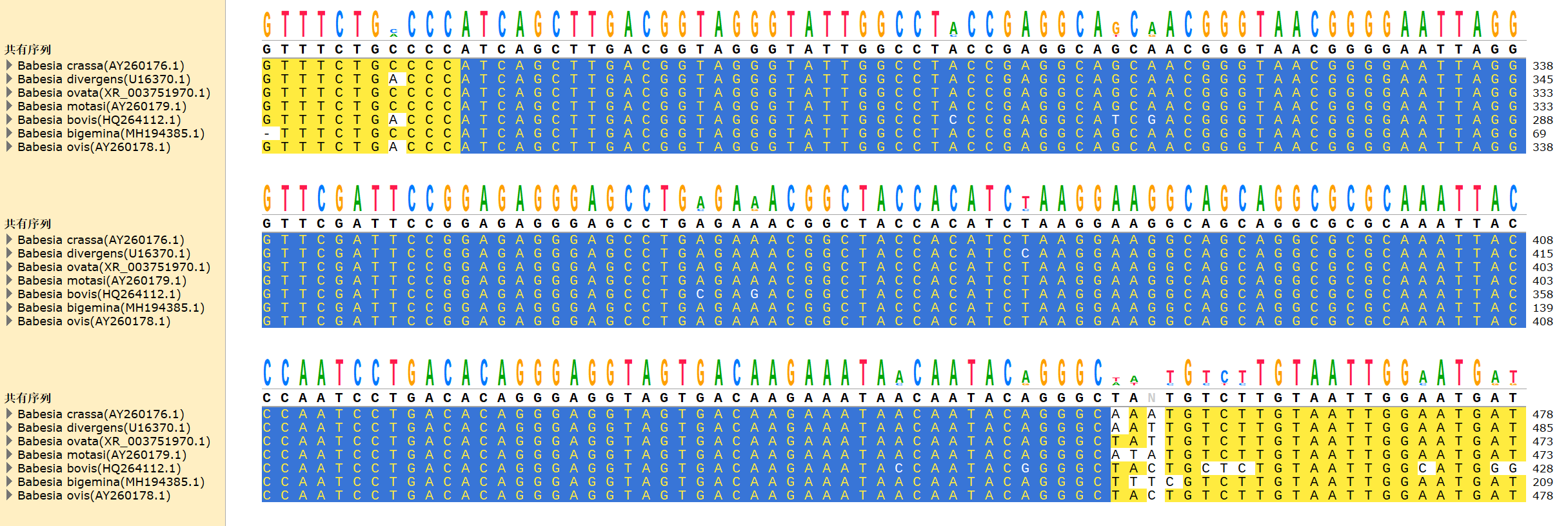


F_IGURE_ S3: Multiple sequence alignment of the 18S rRNA gene from Babesia species infecting cattle and sheep. The 18S rRNA gene sequences of cattle- and sheep-associated *Babesia* species, including *Babesia bigemina* (MH194385.1), *Babesia bovis* (HQ264112.1), *Babesia divergens* (U16370.1), *Babesia ovata* (XR_003751973.1), *Babesia motasi* (AY260179.1), *Babesia ovis* (AY260178.1), and *Babesia crassa* (AY260176.1), were retrieved from GenBank and aligned using SnapGene software. The alignment revealed a highly conserved region within the 18S rRNA gene (blue shading). This conserved region was selected as the target fragment for primer design, providing a basis for broad-range genus-level detection of cattle- and sheep-associated *Babesia* species.

F_IGURE_ S4: Quantitative signal intensity analysis of lateral flow strip results from the specificity assay. Templates representing target *Babesia* spp. and non-target pathogens were tested, including *Babesia*-18S rRNA plasmid, *Babesia bigemina*, *Babesia motasi*, and *Babesia duncani*, as well as non-target pathogens including *Theileria annulata*, *Schistosoma japonicum*, *Trypanosoma evansi*, *Bluetongue virus*, *Leptospira,* *Foot-and-mouth disease virus, Toxoplasma gondii, Brucella melitensis*, and *Neospora caninum*. A no-template control was included in the assay. The heatmap displays the normalized signal intensity of the test line, with the color scale representing signal strength from low intensity in white to high intensity in dark blue.

F_IGURE_ S5: Quantitative signal intensity analysis of lateral flow strip results for the sensitivity assay. Serial dilutions of recombinant plasmid templates ranging from 5 × 10⁶ to 5 × 10^-1^ copies/μL and the no-template control (NTC) were tested. The heatmap displays the normalized signal intensity of the test line, with the color scale representing signal strength from low intensity in white to high intensity in dark blue.


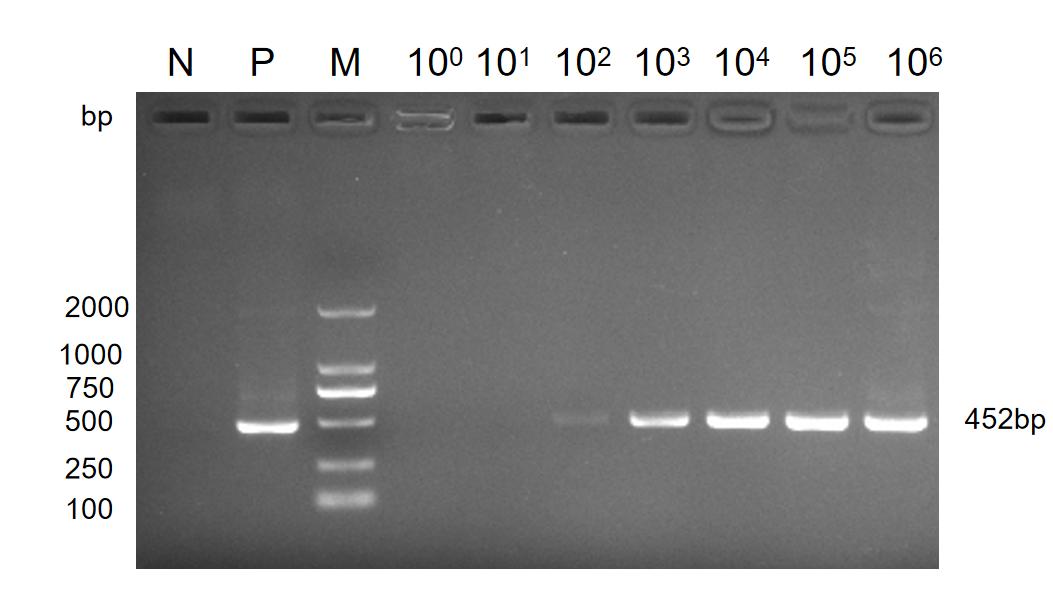


F_IGURE_ S6: Analytical sensitivity evaluation of conventional PCR for the detection of *Babesia* spp. The PCR-specific plasmid standard used for conventional PCR sensitivity analysis was constructed from the target sequence amplified with the conventional PCR primer pair PCR-B-F/R. N, negative control; P, positive control; M, DNA marker; labeled lanes 5 × 10⁰–5 × 10⁶ copies/μL represent ten-fold serial dilutions of the PCR-specific plasmid standard. The expected PCR product size was 452 bp. A specific band was observed from 5 × 10² to 5 × 10⁶ copies/μL, whereas no specific band was detected in the negative control or at lower template concentrations. Therefore, the detection limit of conventional PCR was determined to be 500 copies/μL under the tested conditions.


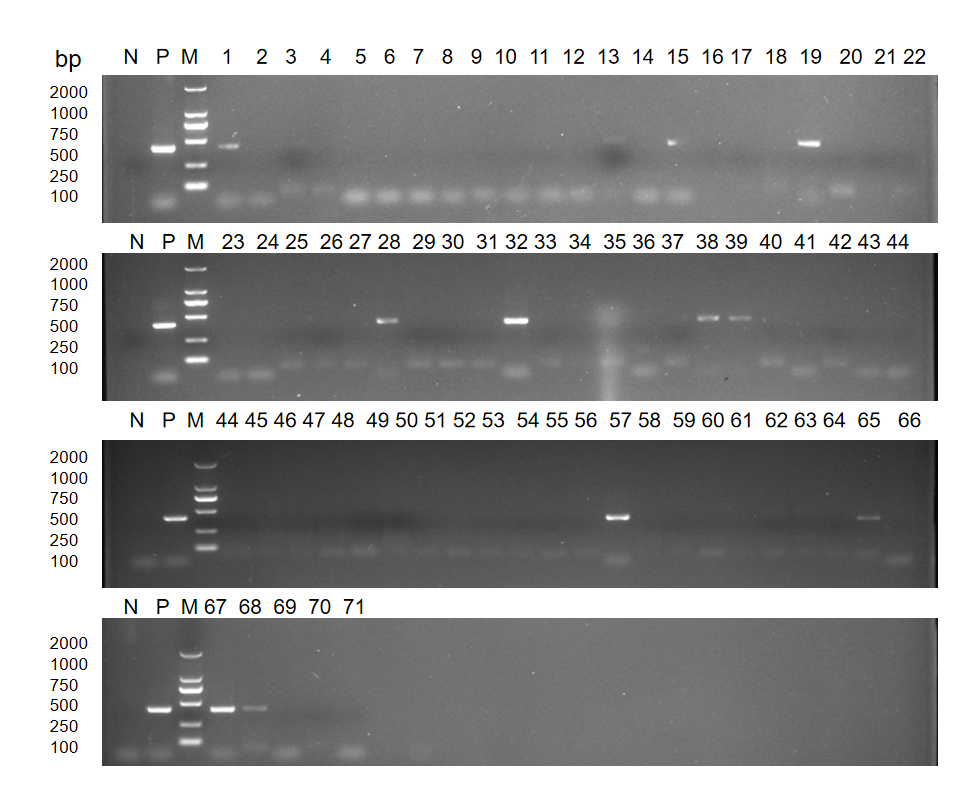


F_IGURE_ S7: PCR and agarose gel electrophoresis were performed on 71 tick sample pools, including pools 1-41 from Shanxi Province and pools 42-71 from Jilin Province.


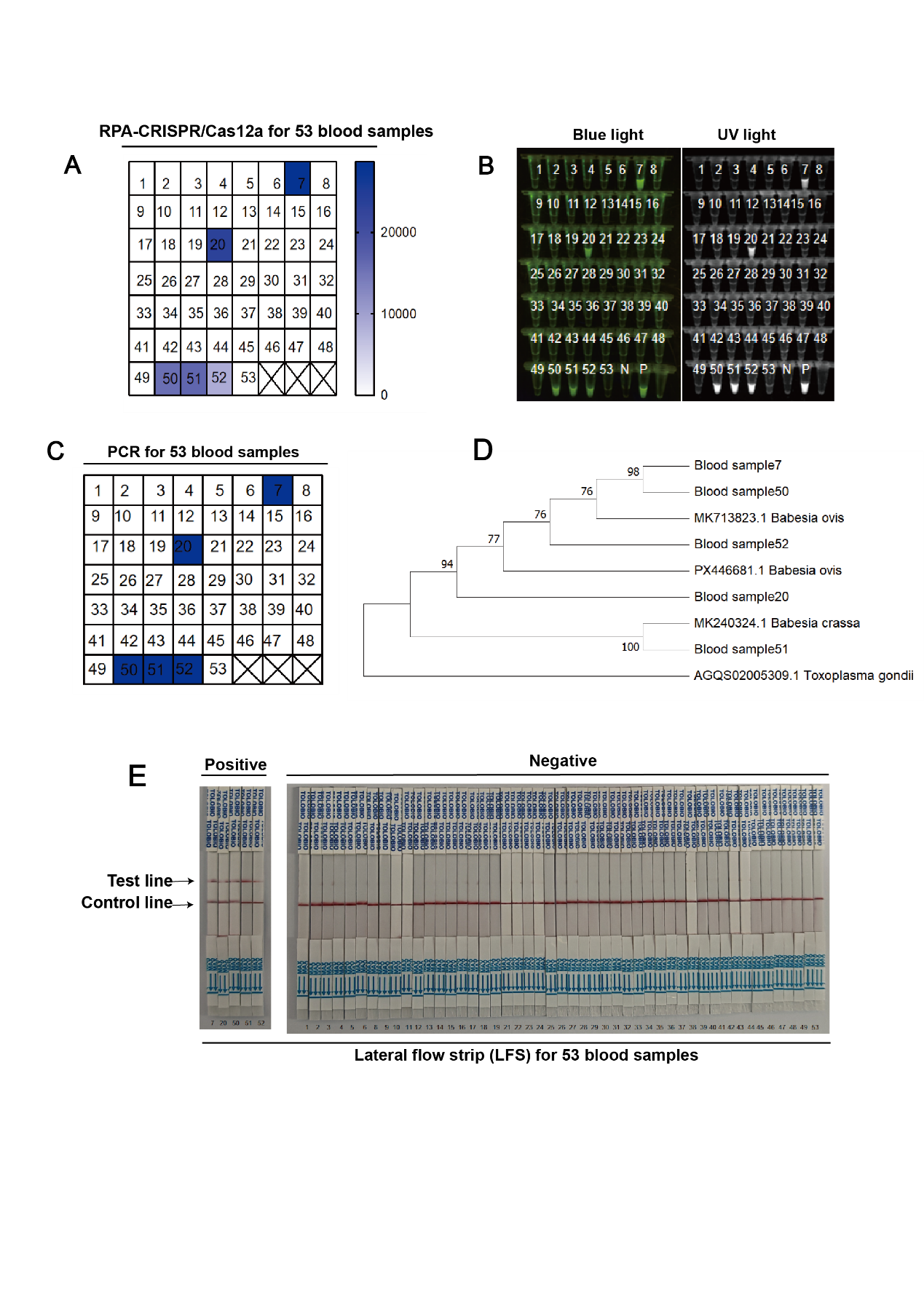


F_IGURE_ S8: Detection and phylogenetic confirmation of *Babesia* spp. in clinical livestock blood samples using the RPA-CRISPR/Cas12a assay. (A) Heatmap analysis of endpoint fluorescence signals obtained from genomic DNA extracted from 53 clinical livestock blood samples using the RPA-CRISPR/Cas12a assay. Color intensity corresponds to fluorescence signal strength, with darker colors indicating positive results and lighter colors indicating negative results. (B) Endpoint fluorescence images of the 53 clinical livestock blood samples captured under blue-light and UV excitation after completion of the RPA-CRISPR/Cas12a reaction. The visual results are fully consistent with the heatmap analysis. (C) Concordance analysis between the RPA-CRISPR/Cas12a assay and conventional PCR for the 53 clinical livestock blood samples. The RPA-CRISPR/Cas12a assay identified the same five positive samples as conventional PCR, showing 100% concordance between the two methods. (D) Phylogenetic analysis of PCR-positive clinical blood sample amplicons after Sanger sequencing of all five positive blood samples. The phylogenetic tree was constructed using the neighbor-joining (NJ) method, with reference sequences of different *Babesia* spp. retrieved from GenBank and *Toxoplasma gondii* used as the outgroup. Four positive blood-derived sequences clustered with *Babesia ovis*, whereas one sequence clustered with *Babesia crassa*. (E) Detection results of the 53 clinical livestock blood samples using LFS based on the RPA-CRISPR/Cas12a assay. Samples showing two distinct bands were interpreted as positive, while those showing only a control band were interpreted as negative.

T_ABLE_ S3: Detection results and diagnostic performance of the assay for 71 pooled tick samples.

| RPA-CRISPR/Cas12a | PCR Positive | PCR Negative | Total |
| --- | --- | --- | --- |
| Positive | 11 | 0 | 11 |
| Negative | 0 | 60 | 60 |
| Total | 11 | 60 | 71 |

Diagnostic indicators: Sensitivity: 100.0%; Specificity: 100.0%; Positive predictive value: 100.0%; Negative predictive value: 100.0%; Overall agreement: 100.0%; Cohen’s Kappa: 1.000.

T_ABLE_ S4: Detection results and diagnostic performance of the assay for 53 clinical livestock blood samples

| RPA-CRISPR/Cas12a | PCR Positive | PCR Negative | Total |
| --- | --- | --- | --- |
| Positive | 5 | 0 | 5 |
| Negative | 0 | 48 | 48 |
| Total | 5 | 48 | 53 |

Diagnostic indicators: Sensitivity: 100.0%; Specificity: 100.0%; Positive predictive value: 100.0%; Negative predictive value: 100.0%; Overall agreement: 100.0%; Cohen’s Kappa: 1.000.


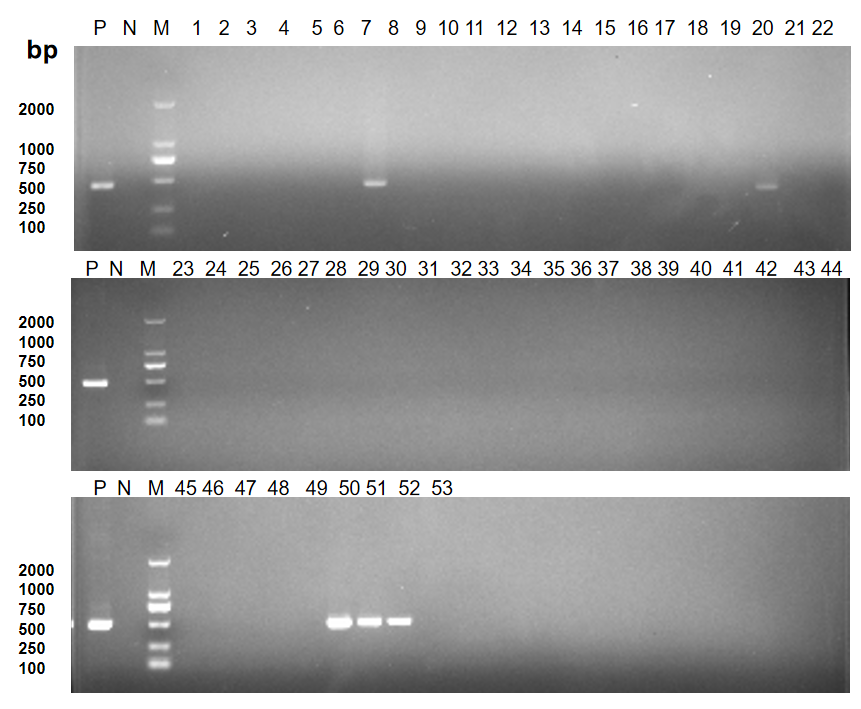


F_IGURE_ S9: PCR and agarose gel electrophoresis were performed on 53 clinical livestock blood samples.


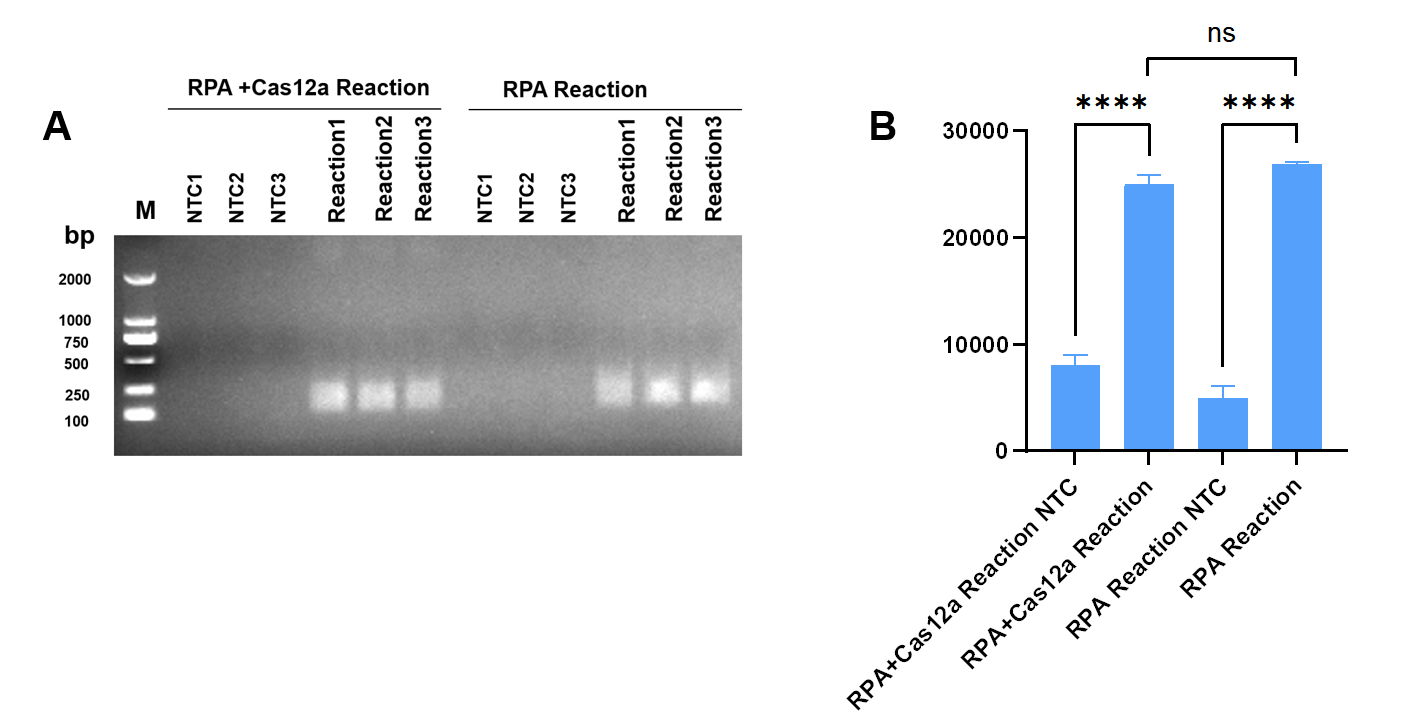


F_IGURE_ S10: Assessment of the effect of Cas12a detection components on RPA amplification. (A) Agarose gel electrophoresis analysis of RPA amplification products generated in the presence or absence of Cas12a detection components. Clear amplification bands were observed in both the RPA with Cas12a components group and the RPA reaction group, whereas no specific bands were detected in the corresponding no-template controls. M, DNA marker. (B) ImageJ-based quantification of amplification-band intensities from the gel shown in (A). Data are presented as mean ± SD from three independent reactions. Both positive reaction groups showed significantly higher band intensities than their corresponding no-template control groups (****p < 0.0001), whereas no significant difference was observed between the RPA with Cas12a components group and the RPA reaction group (ns, not significant). Statistical analysis was performed using one-way ANOVA followed by Tukey’s post hoc test.

T_ABLE_ S5: Descriptive comparison of the present closed-tube, one-pot RPA-CRISPR/Cas12a platform with representative two-step RPA-CRISPR/Cas12a assays.

| Assay/target | Reaction format and handling | Assay time | Signal readout | Reported analytical sensitivity | Comparative features |
| --- | --- | --- | --- | --- | --- |
| *Leptosphaeria maculans* [1] | Two-step workflow. RPA pre-amplification was performed first, followed by manual transfer of RPA products into a separate Cas12a reaction. | 20 min RPA + 20 min Cas12a reaction; approximately 3 min for LFS | Fluorescence and LFS | 4.7 copies/μL by fluorescence; 47 copies/reaction clearly detected by LFS | Provides two readout formats but requires post-amplification product transfer and tube opening. |
| *Pseudomonas aeruginosa* [2] | Two-step workflow. RPA products were transferred to a separate Cas12a detection reaction. | 20 min RPA + 10 min Cas12a reaction | Real-time fluorescence only | Approximately 8 copies/reaction | Rapid but dependent on a real-time fluorescence instrument and lacks endpoint visual or LFS-based interpretation. |
| Deformed wing virus A/B [3] | Two-step workflow. RPA was performed before addition of RPA products to the Cas12a reaction mixture. | 20 min RPA + 20 min Cas12a reaction; 3–5 min for LFD | Endpoint fluorescence and LFD | 6.5 copies/μL for DWV-A and 62 copies/μL for DWV-B | Provides fluorescence and LFD readouts but requires post-amplification transfer; reported sensitivity varies between target variants. |
| Present study: *Babesia* spp. | Closed-tube one-pot workflow. The RPA and CRISPR/Cas12a mixtures were spatially separated before reaction initiation and combined by brief centrifugation within the sealed tube. | 40 min incubation; 3–5 min for LFS | Real-time fluorescence curve monitoring, endpoint fluorescence visualization under blue or UV light, and LFS | 5 copies/μL by fluorescence; 50 copies/μL by LFS | Avoids post-amplification tube opening and supports three result-interpretation formats, providing workflow simplification and flexible application under laboratory and field-oriented conditions. |

Assay times refer to the reported analytical reaction time and generally exclude nucleic acid extraction and, where applicable, reverse transcription or sample pretreatment.

T_ABLE_ S6: Comparison of the established RPA/Cas12a with other molecular detection methods.

| Method | Detection spectrum | Detection limit | Reaction  conditions | Target gene | Time | Reference |
| --- | --- | --- | --- | --- | --- | --- |
| Microscopic examination | *Babesia* spp. in blood samples | / | / | / | 1–2 days | [4] |
| PCR | *Babesia* spp. | 500 copies/µl | / | 18S rRNA gene | 1.5–2 h | This study |
| qPCR | *Babesia spp.* | 5 copies/reaction | / | Mitochondrial LSU rRNA lsu5–lsu4 fragment region | 50 min | [5] |
| qPCR-HRM | Human babesiosis-related species, including *B. microti*, *B. divergens*, *B. duncani* | 1–100 copies/μL depending on species | 55°C | 18S rRNA V3 region | About 1–2 h | [6] |
| Pan-*Babesia* FRET-qPCR | Designed from 22 *Babesia* species; validated for *B. gibsoni*, *B. canis*, *B. vogeli*, *B. microti*, *B. bovis*, and *B. divergens* | About 2 copies/reaction, equivalent to 20 copies/mL whole blood | 58°C | 18S rRNA | About 1–1.5 h | [7] |
| LAMP | Ovine and caprine *Babesia* spp., including *Babesia* sp. BQ1 and *Babesia* sp. Xinjiang-2005 | 0.02 pg genomic DNA for *Babesia* sp. BQ1; 0.2 pg genomic DNA for *Babesia* sp. Xinjiang-2005 | 65°C | 18S rRNA | 60 min | [8] |
| RPA-LFD | Canine *Babesia* spp., mainly *B. vogeli* and *B. gibsoni* | 22.5 copies/μL, approximately 0.1 fg/μL | 40°C | 18S rRNA | 30 min | [9] |
| RPA/CRISPR-Cas12a | Cattle and sheep associated *Babesia* spp. | 5 copies/ul forfluorescence,50 copies/uL for LFSe | 37℃ | 18S rRNA | 40 min | This study |

**Reference**

[1] R. Lei, Y. Li, L. Li, et al.,"A CRISPR/Cas12a-based portable platform for rapid detection of Leptosphaeria maculans in Brassica crops."*Front Plant Sci* 13,no.2022):976510.

[2] S. Liu, S. Huang, F. Li, et al.,"Rapid detection of Pseudomonas aeruginosa by recombinase polymerase amplification combined with CRISPR-Cas12a biosensing system."*Front Cell Infect Microbiol* 13,no.2023):1239269.

[3] Y. Xiao, D. Fei, M. Li, et al.,"Establishment and Application of CRISPR-Cas12a-Based Recombinase Polymerase Amplification and a Lateral Flow Dipstick and Fluorescence for the Detection and Distinction of Deformed Wing Virus Types A and B."*Viruses* 15,no.10(2023):

[4] P. J. Krause, P. G. Auwaerter, R. R. Bannuru, et al.,"Clinical Practice Guidelines by the Infectious Diseases Society of America (IDSA): 2020 Guideline on Diagnosis and Management of Babesiosis."*Clin Infect Dis* 72,no.2(2021):e49-e64.

[5] B. A. Qurollo, N. R. Archer, M. E. Schreeg, et al.,"Improved molecular detection of Babesia infections in animals using a novel quantitative real-time PCR diagnostic assay targeting mitochondrial DNA."*Parasit Vectors* 10,no.1(2017):128.

[6] J. Wang, A. Liu, S. Zhang, et al.,"High resolution melting analysis of the 18S rRNA gene for the rapid diagnosis of bovine babesiosis."*Parasit Vectors* 12,no.1(2019):523.

[7] J. Li, P. Kelly, J. Zhang, et al.,"Development of a pan-Babesia FRET-qPCR and a survey of livestock from five Caribbean islands."*BMC Vet Res* 11,no.2015):246.

[8] G. Guan, A. Chauvin, J. Luo, et al.,"The development and evaluation of a loop-mediated isothermal amplification (LAMP) method for detection of Babesia spp. infective to sheep and goats in China."*Exp Parasitol* 120,no.1(2008):39-44.

[9] W. Onchan, O. Ritbamrung, P. Changtor, et al.,"Sensitive and rapid detection of Babesia species in dogs by recombinase polymerase amplification with lateral flow dipstick (RPA-LFD)."*Sci Rep* 12,no.1(2022):20560.
